# Hot weather hazard analysis over India

**DOI:** 10.1038/s41598-022-24065-0

**Published:** 2022-11-17

**Authors:** Akhil Srivastava, M. Mohapatra, Naresh Kumar

**Affiliations:** India Meteorological Department, Ministry of Earth Sciences, Government of India, New Delhi, India

**Keywords:** Natural hazards, Atmospheric dynamics

## Abstract

Heat waves are often termed as the silent killer and have become even more important as recent studies suggest that the heat wave have become second most devastating extreme weather events in terms of human deaths and losses. It is also been largely realised by scientific community that it is not just the high temperatures which are responsible for the gruesome effect of heat waves but several other meteorological parameters play a vital role in aggravating the impact and causing much more damages. In view of the above the attention of scientific community, weather forecasters as well as disaster managers has shifted to also take into account the different meteorological parameters like maximum and minimum temperatures, relative humidity, wind speed, duration/spell of heat waves and its intensity which are aggravating the impact of heat stress. In this background, this study is undertaken as an attempt to quantify the effect of different meteorological parameters on heat wave on different regions of India for different summer months (March, April, May and June). In this study the impact of individual meteorological parameter as well their cumulative effect is studied based on data of 30 years (1981–2010) for 300 stations. The effect of different meteorological parameters is identified for different months for different regions of the country. Also the cumulative scores are calculated for different regions considering different meteorological parameters, as a first initiative to perform heat hazard analysis and zonation over the entire country. This could serve as initial step for planning mitigation and adaptation strategies throughout the country. These scores as thresholds for different regions may be also useful for operational forecaster’s for early impact based warning services as well as for the disaster managers, for taking effective and timely actions.

## Introduction

Indian sub-continent faces various extreme weather events (EWE) leading to loss of life and property. This EWE include tropical cyclones, floods, heavy rainfall, heat waves, cold waves, landslides, and thunderstorm and lightning etc. and they affect different parts of the country during different months of the year^1–6^. There has been lot of studies to understand and reduce the losses due to these EWE’s by identifying their spatial and temporal occurrences and by doing vulnerability mapping of these EWE’s. There has been particularly lot of improvement in early warning of cyclones which helped to reduce losses due to Tropical Cyclones, which was leading cause of deaths among all EWE’s^2,7,8^. Similarly, spatial and temporal heat hazard zonation becomes important to identify and reduce the loss of lives due to heat wave events. This becomes even more important due to the various studies suggesting increase in surface air temperature and increased frequency of heat waves attributing them to Climate Change over India^9,10^.

Heat waves events number have increased by about 24% during 2010–2019 as compared to 2000–2009 and associated mortality rates have increased by about 27% transforming Heat waves, from the third most devastating EWE responsible for mortalities to second most disastrous event linked to mortality^11^. The mortality rate of tropical cyclones reduced by 94% in the past 20 years, whereas for heat-waves it increased by 62.2%^11^.

Heat waves in India are expected to intensify and cause increase in heat stress. To mitigate heat induced mortality requires preparedness as well as adaptation measures. The limited resources available for the mitigation and adaptation measures need to be well directed. For this purpose it is required to identify the areas where heat stress leads to more mortalities and losses and channelize the resources to these areas to save maximum lives. The major difficulty in this identification is to understand the phenomenon which aggravates the impact of heat waves and make them dangerous for survival of human being.

The World Meteorological Organization (WMO) defines Heat Waves as a period of prolonged abnormally high surface temperatures relative to those normally expected. Such periods with maximum temperatures exceeding 5 °C above normal and persisting for 5 consecutive days are termed as Heat Waves as per WMO (https://www.britannica.com/science/heat-wave-meteorology); According to United States National Weather Service (US-NWS) “a heat wave is a period of abnormally hot weather generally lasting more than 2 days. Heat waves can occur with or without high humidity”. Australia Bureau of Meteorology defines the criteria of heat wave as to “when the maximum and the minimum temperatures are unusually hot over a 3-day period at a location. This is considered in relation to the local climate and past weather at the location”. In Denmark, a national heat wave is defined when for at least three consecutive days average maximum temperature in more than half of the country exceeds 28 °C. Sweden uses the criteria that when for at least five consecutive days daily maximum temperature exceeds 25 °C, then heat wave is declared. India Meteorological Department defines heat waves and severe heat waves as per following criteria.

“Heat wave is considered if maximum temperature of a station reaches at least 40 °C or more for Plains, at least 37 °C for Coastal region and at least 30 °C or more for Hilly regions. The heat wave are defined as follows.

- Based on departure from normal
  (i) Heat wave: Departure from normal is 4.5 °C to 6.4 °C.
  (ii) Severe heat wave: Departure from normal is > 6.4 °C.
- Based on actual maximum temperature:
  (i) Heat wave: When actual maximum temperature ≥ 45 °C.
  (ii) Severe heat wave: When actual maximum temperature ≥ 47 °C.

If above criteria met at least in 2 stations in a Meteorological sub-division for at least two consecutive days and it declared on the second day”^12^.

Therefore, it is widely seen that the heat wave criteria are not uniform across the globe and there are local and regional influences while defining the heat wave. Many studies conducted by researchers are based on different criteria’s of heat wave and also different other meteorological parameters are considered while concluding its impact over people. There was a study on heat wave over Europe where the criteria was that for three consecutive days in summer the daily maximum temperature should have crossed the 80th percentile of daily maximum temperature^13^ and in another study over urban areas of globe the daily maximum temperature exceeding 99th percentile of maximum temperature for consecutively 6 days or more was considered^14^. Similarly, in one of the studies over India to investigate heat waves maximum temperature departure by 3 °C or more than the normal temperature consecutively for 3 days or more was considered^15^.

Above mentioned studies and most of the research primarily focussed on the single temperature metric (e.g., daily mean/minimum/maximum temperature) based heat wave and their impacts due to the reason that Temperature measurements are near ubiquitous^16^. Studies have also concluded that there exists large differences in the best temperature measure between age groups, seasons and cities, and there was no one temperature measure that was superior to the others^16^. The strong correlation between different measures of temperature means that, on average, they have the same predictive ability and best temperature measure can be chosen based on practical concerns^16^. The mechanism by which heat impacts humans is complex; it is a result of the interactions between temperature, radiation, wind, and humidity^17–19^. There is strong experimental evidence that physiologic stress from high temperatures is greater if humidity is higher^20,21^. However, heat indices developed to allow for this have not consistently predicted mortality better than dry-bulb temperature^20^. Further few studies have demonstrated that metrics which are thought to be better predictors of physiological heat stress by considering several weather conditions simultaneously may not be better at predicting heat-related mortality, which has significant implications in heat wave and health warning systems^22^.

Few of the studies have been conducted specifically for India to understand the heat wave, its reasons, impact on human life and predictability^11,23–29^. There are different conditions/processes which play an important role in modulating the effect of heat waves. In a study impact of intensive irrigation is found to cools the land surface by 1 °C and the air by 0.5 °C. However, the decreased sensible heat flux due to irrigation reduces the planetary boundary layer height, which increases low-level moist enthalpy. Thus, irrigation increases the specific and relative humidity, which raises the moist heat stress metrics. Intense irrigation over the region results in increased moist heat stress in India, Pakistan, and parts of Afghanistan^27^. Similarly in a study on heat stress over Delhi, Kolkata, Mumbai and Chennai based on daily maximum temperature, relative humidity, wind speed and solar radiation datasets during 1990–2019 found the varying risk of heat stress on these regions. Delhi witnesses lower heat stress because of lower RH although temperature is higher in this region. While Delhi experiences more heat waves, the risks of heat stress and dangerous-heat stroke events are maximum in Chennai due to high temperature along with higher RH. The risk of extreme heat stress is lower in Mumbai region because of relatively lower temperature than Chennai during summer season. The likelihood of experiencing great discomfort during heat wave periods in Kolkata city is higher than that experienced in other metropolitan cities in India. However, during non-heat wave periods the probability of extreme discomfort is higher in Chennai^28^. In another study, the estimated heat stress is found to have more impact on the coastal areas of India having exposure to more frequent days of extreme heat stress conditions along with the increased probability of occurrence. The explicit amount of change in temperature, increase in the duration and intensity of warm days along with the modulation in large scale circulation in future are seemingly connected to the increasing levels of heat stress over India^29^. A decline of 30 to 40% in the work performance is projected over India by the end of the century due to the elevated heat stress levels which pose great challenges to the country policy makers to design the safety mechanisms and to protect people working under continuous extreme hot weather conditions^29^. Few of the past studies have dealt with the Heat Wave impact utilising the Heat Index and other similar indices^21^, however, these indices have a limitation in terms of their applicability based on different thresholds. For example the studies using heat index equation based on the Steadman’s equation^17,18^ are limited by their validity on the regions when air temperature and relative humidity are higher than 80 °F (26 °C) and 40%, respectively. However, these are not climatologically found to be valid over central, northwest and other interior parts of India during summer/pre-monsoon seasons^30–32^.

## Characteristics of heat waves over India

Indian subcontinent experiences summer (pre-monsoon) season between March to May (as per the nomenclature of India Meteorological Department), and most of the land part is hottest during May due to direct solar heating from transiting Sun towards north, with heat accumulation arising from desert in the northwest parts and physiography of the central plateau and northern plains^33^. The extended events of heat Wave may last up to June for regions where southwest monsoon has not reached. Therefore, climatologically heat waves are seen over India during March to June^21,24^ with high frequency over north, northwest, central and the eastern coastal regions of India^9^. Some studies have also suggested that over central and northwestern parts of the country, frequency, total duration and maximum duration of heat waves and their impacts are increasing^5,21^.

India witnesses two types of heat waves: The first-type of heat wave over the north-central India is found to be associated with blocking over the North Atlantic causing heat wave conditions over India and the second-type of heat wave over the coastal eastern India is found to be due to the anomalous Matsuno-Gill response to the anomalous cooling in the Pacific^24^. The heat waves over India have been linked with the climate modes such as El Niño-Southern oscillation (ENSO)^34^. Some studies also linked the heat waves to the variations in the sea surface temperatures in the Bay of Bengal and re-curving tropical cyclones in the Bay of Bengal^35^. As far as mortality is concerned, detailed statistical comparison was carried out in a study for two 20-year slice periods, i.e., 1980–1999 and 2000–2019. It showed an increase of 138% in the heat wave, with the mortality rates per million for heat waves have increased by 62.2%^11^.

Post 2015, heat waves studies got more interest and motivation after Heat waves were notified as Disaster at national level by National Disaster management Authority, India. Also 2015 saw worst Heat wave impact over India with more than 2000 lives lost due to Heat Waves. The number of Indian states getting affected by Heat Waves have climbed from 9 in 2015 to 23 in 2020^36^. This led to more focussed studies on Heat Waves with special interest to identify the region wise meteorological parameters which could help to figure out the impact of heat waves and help in reducing the mortality associated with the heat wave events.

In this respect, present study aims to provide a Heat Hazard Zonation map of India considering the different meteorological parameters. The study tries to bring out the share of different meteorological parameters role in aggravating the impact of heat waves in different parts of the country. The effort is to bring out month wise climatological daily heat hazard scores which could help in identifying the most vulnerable areas with respect to different meteorological parameters for effective adaptation and mitigation planning. Forecasters can utilize these scores as a threshold and guidance for identification of heat hazard days in different regions of India considering the impact of different meteorological parameters in short to medium temporal range (1–10 days).

### Data

**Figure 1:**
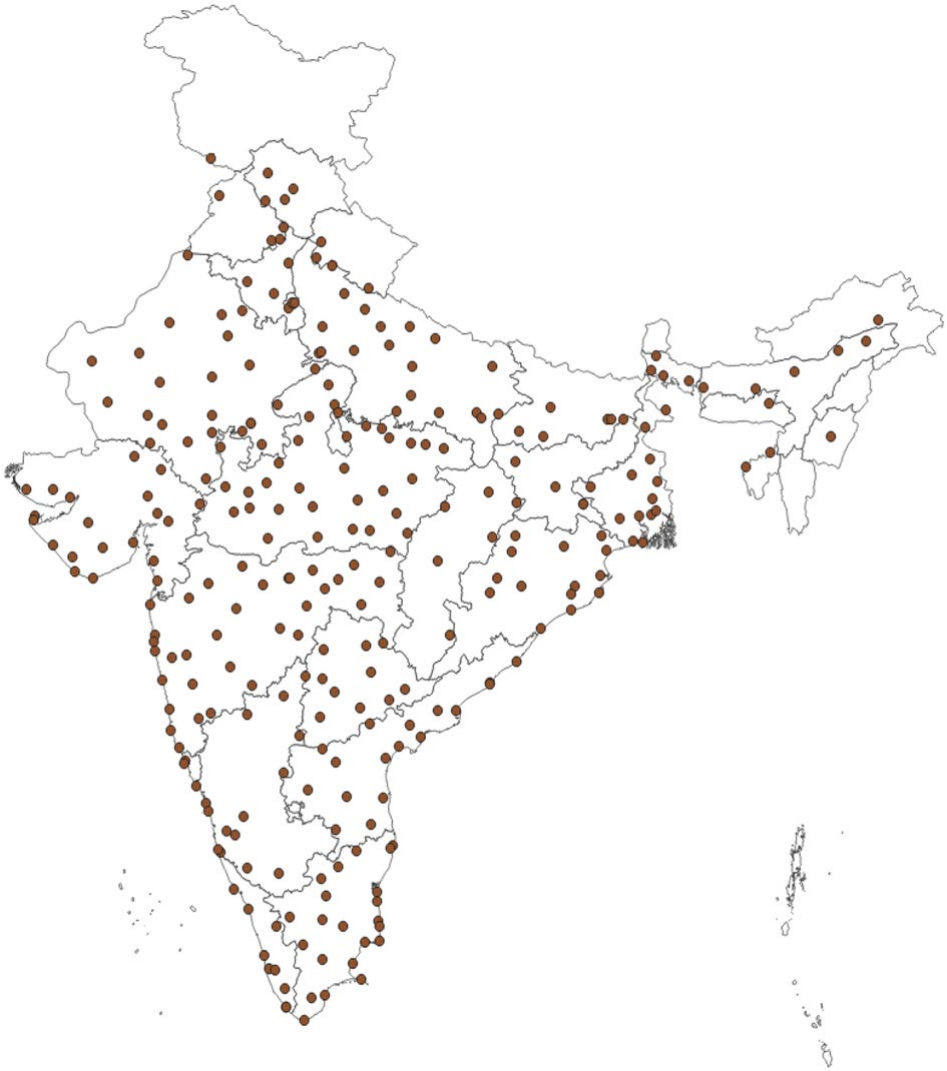
Stations under consideration in this study.

There has been multitude of methods being used across the globe to quantify heat wave and its impact. The most important among them is the Air Surface temperature or the Dry Bulb temperature. In few studies Heat Wave is also influenced based on the Minimum Temperatures or by combining the Maximum and Minimum temperatures. Quantifying impact of heat waves is complex as it involves heat transfer between human body and environment^37^. It becomes even more complex as the impact of heat waves can be due to a single variable, such as extreme temperature, or to a combination of variables not all of which are necessarily extreme^38^. Many attempts have been made to express human heat stress as function of air temperature, humidity, duration and wind speed etc. In humid regions, high humidity also becomes very important in estimation of severity of heat waves. Humans’ ability to efficiently handle wet-bulb temperature of 35 °C marks our upper physiological limit, and much lower values have serious health and productivity impacts. Therefore while magnitude, duration and frequency of heat waves are of general importance to estimate impact of heat waves but it would miss out on the information provided by other factors such as humidity and wind speed. Therefore, in this study different meteorological parameters are taken into consideration for estimating the mean daily spatial heat hazard scores over India for months of March, April, May and June. For the present study the surface maximum temperatures, minimum temperatures, 09 UTC relative humidity and 09 UTC wind speed is considered for various field stations of IMD for the duration of 30 years (1981 to 2010). As the IMD’s heat waves classification criteria are different for Plain stations, Hilly Stations and Coastal Stations therefore as a first step, the 300 stations are distinguished as Plain Stations, Hilly Stations and Coastal Stations. The stations at the height of 500 m and above of mean sea level are considered as Hilly Stations for this study. The stations which are within 50 km from the sea coast are considered as Coastal Stations and the rest of the stations are considered as Plain Stations. Out of 300 stations, 203 stations are Plains, 39 are Hilly and 58 are Coastal Stations. The stations under considerations are distributed evenly throughout the country. Daily surface maximum and minimum temperature and wind as well as Relative Humidity data required for calculating heat hazard analysis are taken from National Data Centre (NDC), India Meteorological Department (IMD), and Pune. NDC uses a series of robust quality control methods before archiving the data^39^. The doubtful data is filtered out at the initial stages by manual checking and then they are further subjected to quality control procedures at NDC to eliminate any spurious values. Geographical locations of these stations are shown in Fig. 1.

## Methodology and results

**Figure 2:**
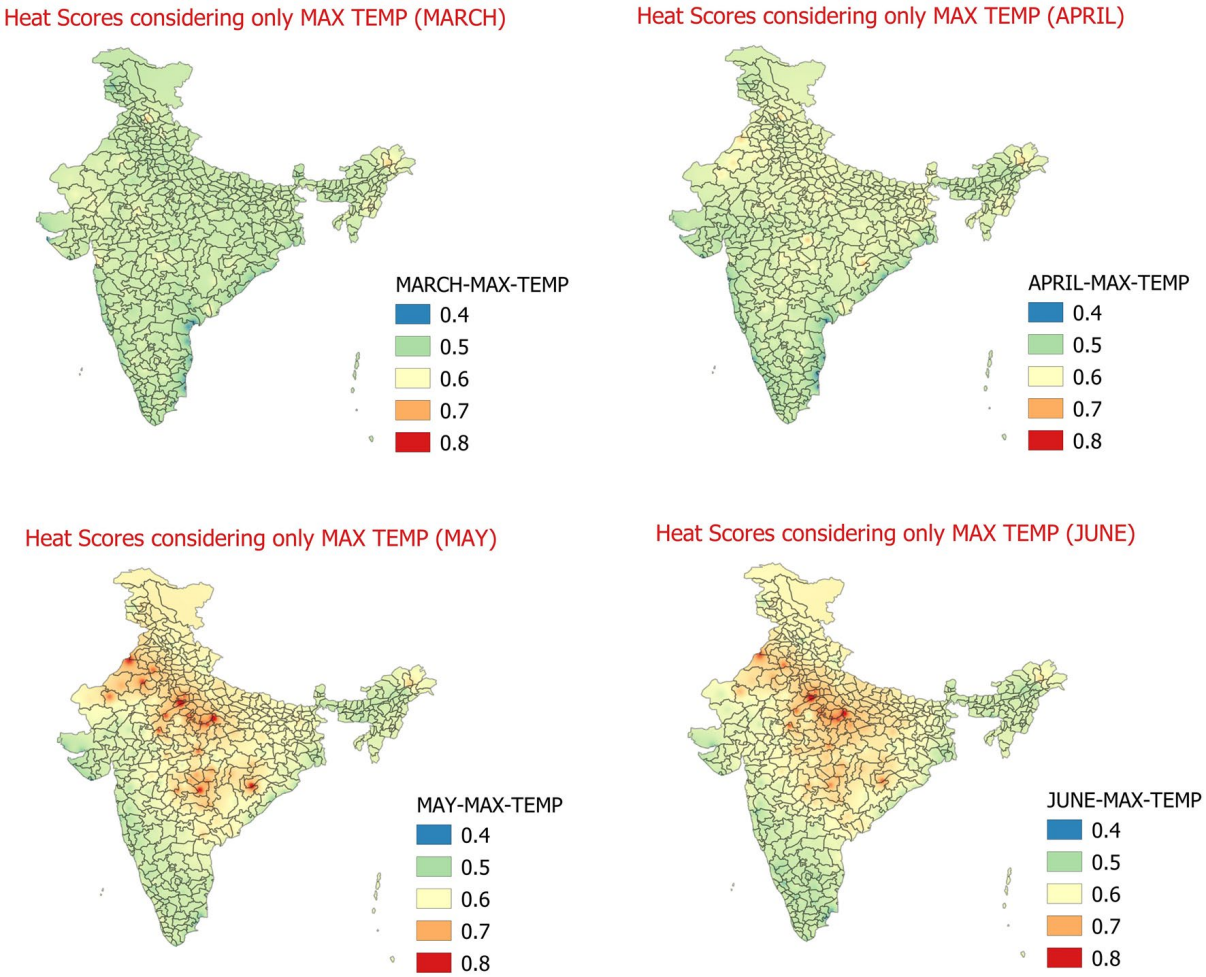
Daily average heat hazard scores considering only max temperature March (Top-left), April (Top-Right), May (Bottom-left), June (Bottom-right).

The study is performed in the following steps as detailed below by generating maps using QGIS software^40^: 1st step takes into account the heat hazard estimation which is generated based on Maximum temperature & their departures as per definitions of heat wave given by India Meteorological Department. In this step the heat wave, severe heat wave and above normal maximum temperature days are identified using IMD’s criteria for Plain stations, Hilly Stations and Coastal Stations from the available set of data (1981–2010) for 300 stations covering India. In this way, for each of the stations we have the above normal maximum temperature days, heat wave and severe heat wave days for March to June months of 1981 to 2010. The weight of 1 is given to each day when maximum temperatures are above normal, weight of 2 is given when maximum temperatures of a day meets IMD’s Heat Wave criteria and weight of 3 is given when maximum temperature of a day meets IMD’s severe heat wave criteria. The days with normal and below normal maximum temperatures are given 0 weights. These scores are then aggregated for each month (March to June) for each station. Finally average daily scores for each of the months of March to June are calculated. The daily average weight scores obtained are shown below in Fig. 2.

In the 2nd step Heat hazard scores are estimated based on minimum temperature and their departures from normal for all the days when maximum temperatures were found to be above normal or were meeting heat wave or severe heat wave criteria. For this purpose, similar to step 1, the days with above normal minimum temperatures and days meeting the IMD’s criteria of warm nights and severe warm nights are identified from the available set of data (1981–2010) for 300 stations. The warm night and severe warm night is defined based on departures of minimum temperatures from normal minimum temperature of a station as given below.

- Warm night: minimum temperature departure is 4.5 °C to 6.4 °C
- Very warm night: minimum temperature departure is > 6.4 °C

**Figure 3:**
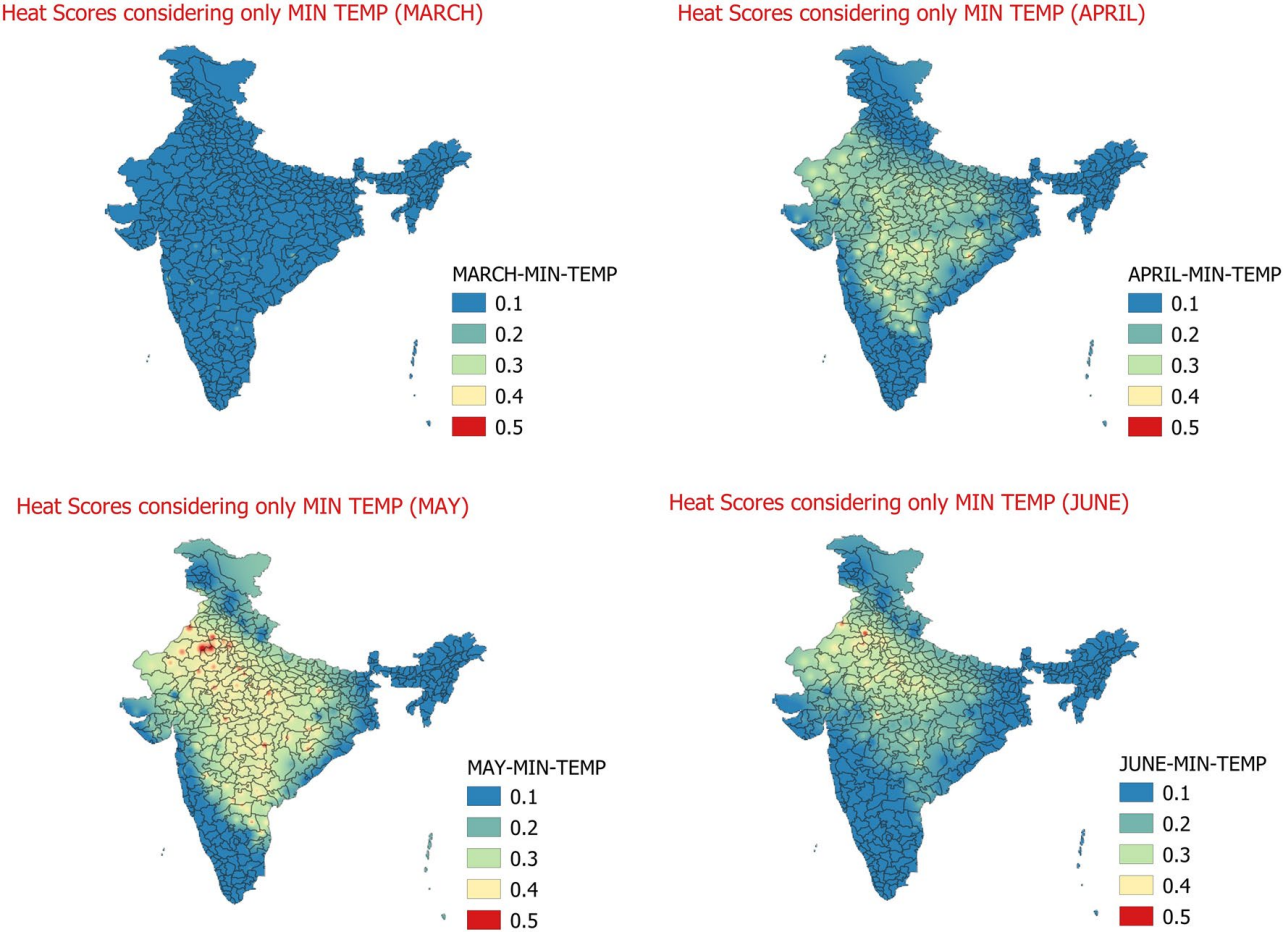
Daily average heat hazard scores considering only min temperature March (Top-left)﻿ , April (Top-Right)﻿, May (Bottom-left)﻿, June (Bottom-right)﻿.

In this step, for each of the 300 stations we have the information of above normal minimum temperature days, days meeting Warm nights and Severe Warm night criteria for March to June months of 1981 to 2010. Weights of 0 is given to days with normal or below normal minimum temperatures, weight of 1 is assigned to days which have reported above normal minimum temperatures, weight of 2 is assigned to days with warm nights and weight of 3 is given to days with severe warm nights. These scores are then aggregated for each month for each station. Finally average daily scores for each of the months from March to June are calculated. The daily average weight scores obtained are shown below in Fig. 3.

In the 3rd step, the estimation of the effect of relative humidity in aggravating the impact of heat wave is attempted. For this purpose, the relative humidity (RH) of 09 UTC is obtained from IMD for 158 stations under consideration for months of March, April, May and June for duration of 1981–2010. The idea is to include the effect of excess humidity above what is normal to be included in the estimate of heat hazard for all the days when maximum temperatures are above normal. Now as few of the stations under consideration are coastal and few are plains and hilly. In this situation the normal RH for plains would be very small as compared to coastal stations and there would be more score bias of RH towards these stations. To overcome this problem the average RH for all the coastal stations in the east coast of India for months of March to June is obtained for the duration of 1981–2010. This average RH value for each month is taken as the lowest limit or threshold of RH for assigning any weights to the RH. The monthly average RH values from all the east coast stations is given in Table 1.

**Table 1:** Monthly average Relative Humidity (RH) values (09UTC) from the East coastal stations of India.

Month	Average RH (East Coast)
March	60.35
April	62.63
May	61.39
June	61.19

**Figure 4:**
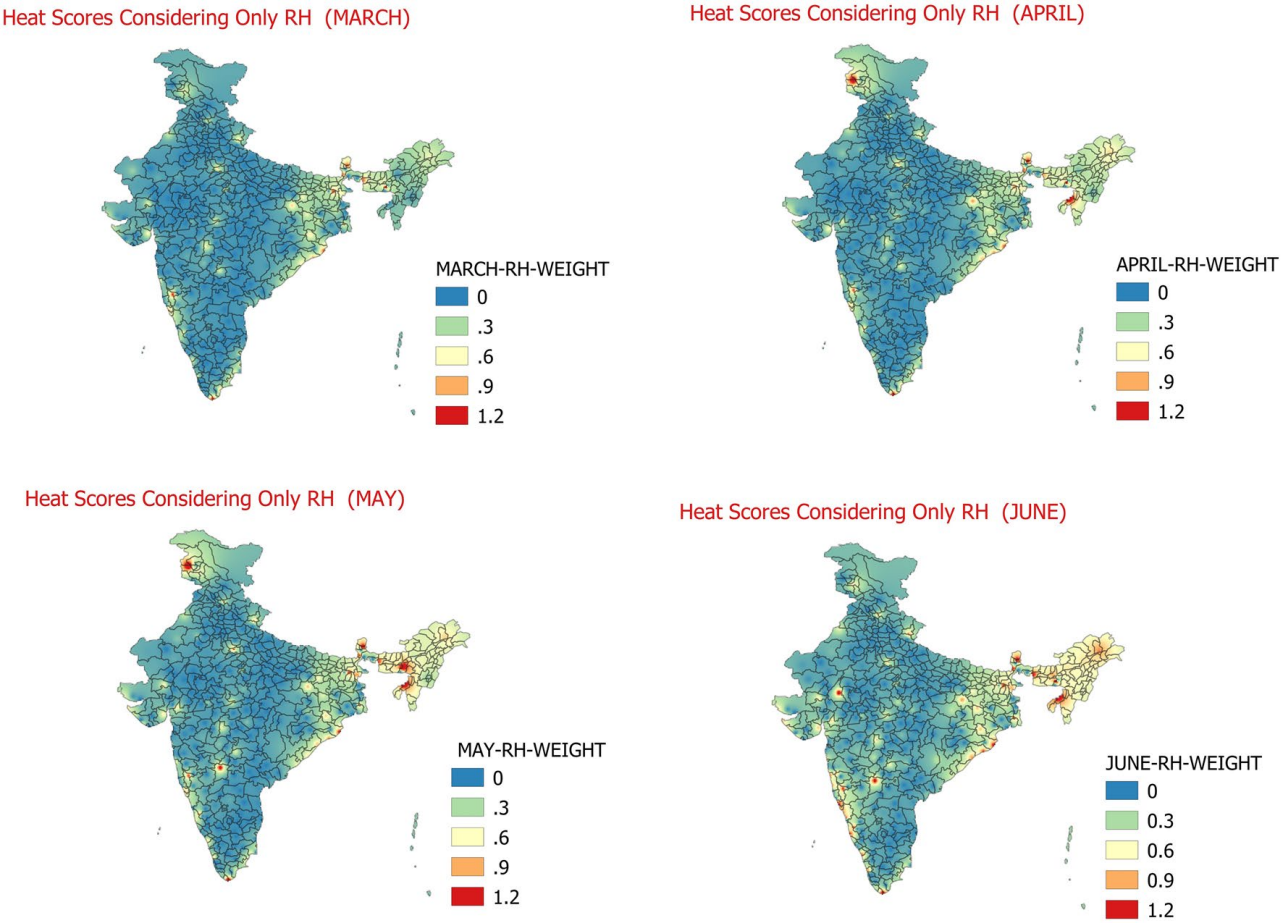
Daily average heat hazard scores considering only RH at 09 UTC for March (Top-left)﻿, April (Top-Right)﻿, May (Bottom-left)﻿, June (Bottom-right)﻿.

The normal RH of each of the 158 stations is calculated for each of the months of March to June for duration of 1981 to 2010. The maximum RH value of each station for the months of March to June during 1981 to 2010 is identified from the data. The maximum of average RH as obtained from east coast stations and normal RH identified from data for 158 stations is taken as RH threshold for that particular station for the respective month. The RH values between threshold RH and the maximum RH are then divided into 3 equal parts and weights of 1, 2 and 3 respectively are assigned to them (RH_A, RH_B, and RH_C) for each month of March to June during 1981 to 2010. The weight of 0 (RH_D) is assigned to any day when the RH is below Threshold RH during the period 1981 to 2010. The RH for each station in the month of March to June during 1981–2010 with above normal maximum temperatures days, and days meeting IMD’s Heat Wave day or Severe Heat Wave criteria is matched with the RH bands of RH_A, RH_B, RH_C and RH_D. In which ever band the RH values for the identified day lies, corresponding to that band the weightage of RH is assigned to that day. The weights of RH are then added for each month for all the days and monthly daily average scores of RH for March to June months are calculated. The daily average RH weight scores for March to June months are shown below in Fig. 4.

**Figure 5:**
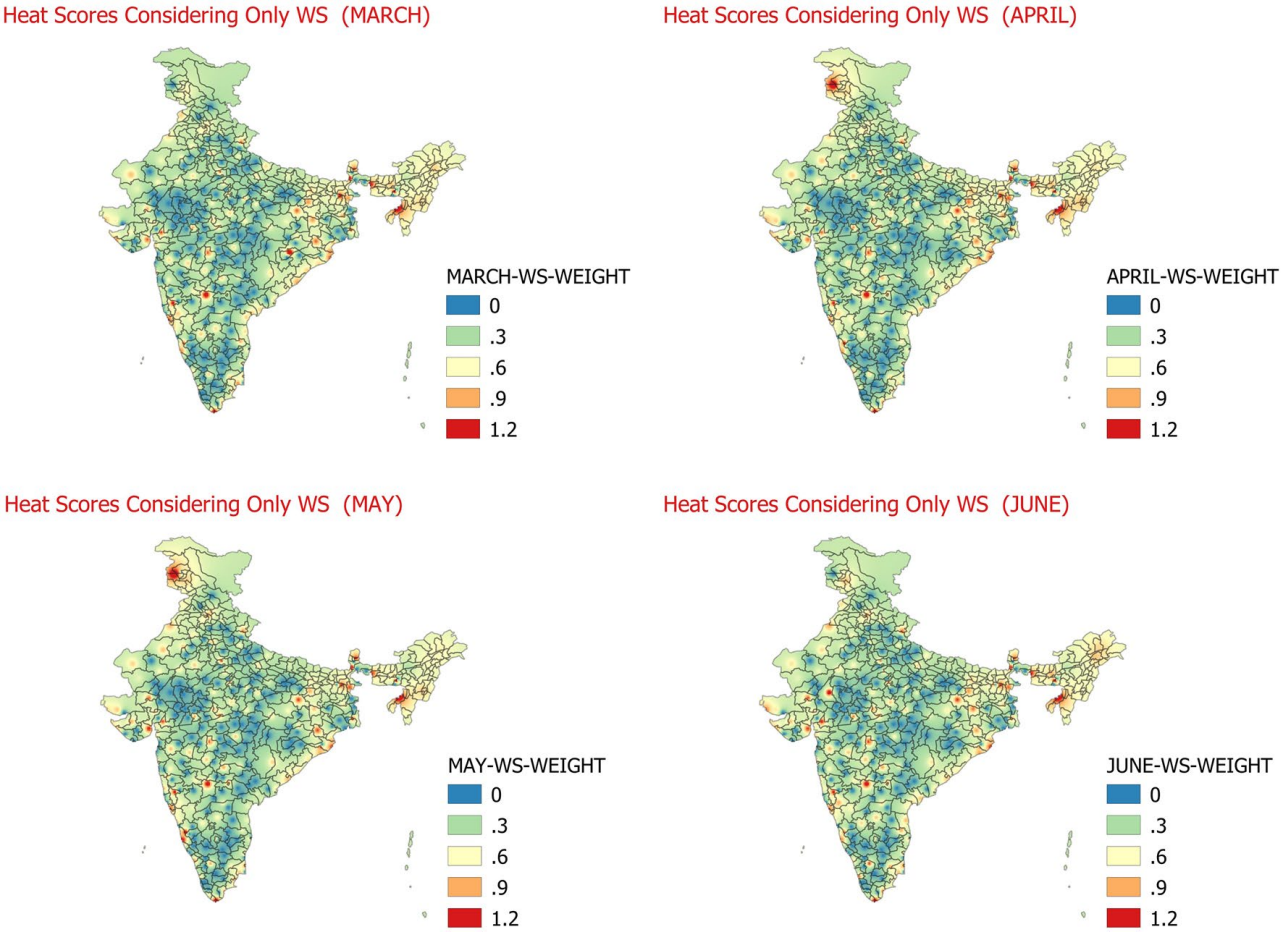
Daily average heat hazard scores considering only WS at 09 UTC for March (Top-left)﻿, April (Top-Right)﻿, May (Bottom-left)﻿, June (Bottom-right)﻿.

In the 4th step, the estimation of individual relative effect of wind speed (WS) is attempted on the impact of heat waves. For this purpose, the 09 UTC wind speed (WS) is obtained for 158 stations under consideration for months of March, April, May and June for duration of 1980–2010. The threshold for wind speed is taken as 02 knots. The Wind speed average (normal WS) individually for all the days of the month during 1981–2010 at 09 UTC for these stations under consideration is calculated from the data. If this normal wind speed is greater than the threshold wind speed of 02 knots then the threshold wind speed is replaced with normal WS. The maximum WS value at 09 UTC of each station is also identified from the data for all the years for months of March to June. The Threshold WS and the maximum WS for any station in the month of March to June is partitioned into three bands WS_A, WS_B and WS_C having weightage of 1, 2 and 3 respectively. The days with 09 UTC wind speed less than threshold wind speed are assigned 0 (WS_D) weights. The WS for each station in the month of March to June during 1981–2010 with above normal maximum temperatures days, and days meeting criteria of IMDs heat wave day or severe heat wave day is matched with the WS bands of WS_A, WS_B, WS_C and WS_D. In which ever band the WS values at 09 UTC for the above normal maximum temperature days, and days meeting criteria of IMDs heat waves or severe heat wave day lies, corresponding to that band the weightage of WS is identified for that particular day. These scores for all the days of a month are then aggregated and daily average weights of wind speed for month in March to June are calculated. The month wise daily average WS weight scores for each month are shown below in Fig. 5.

**Figure 6:**
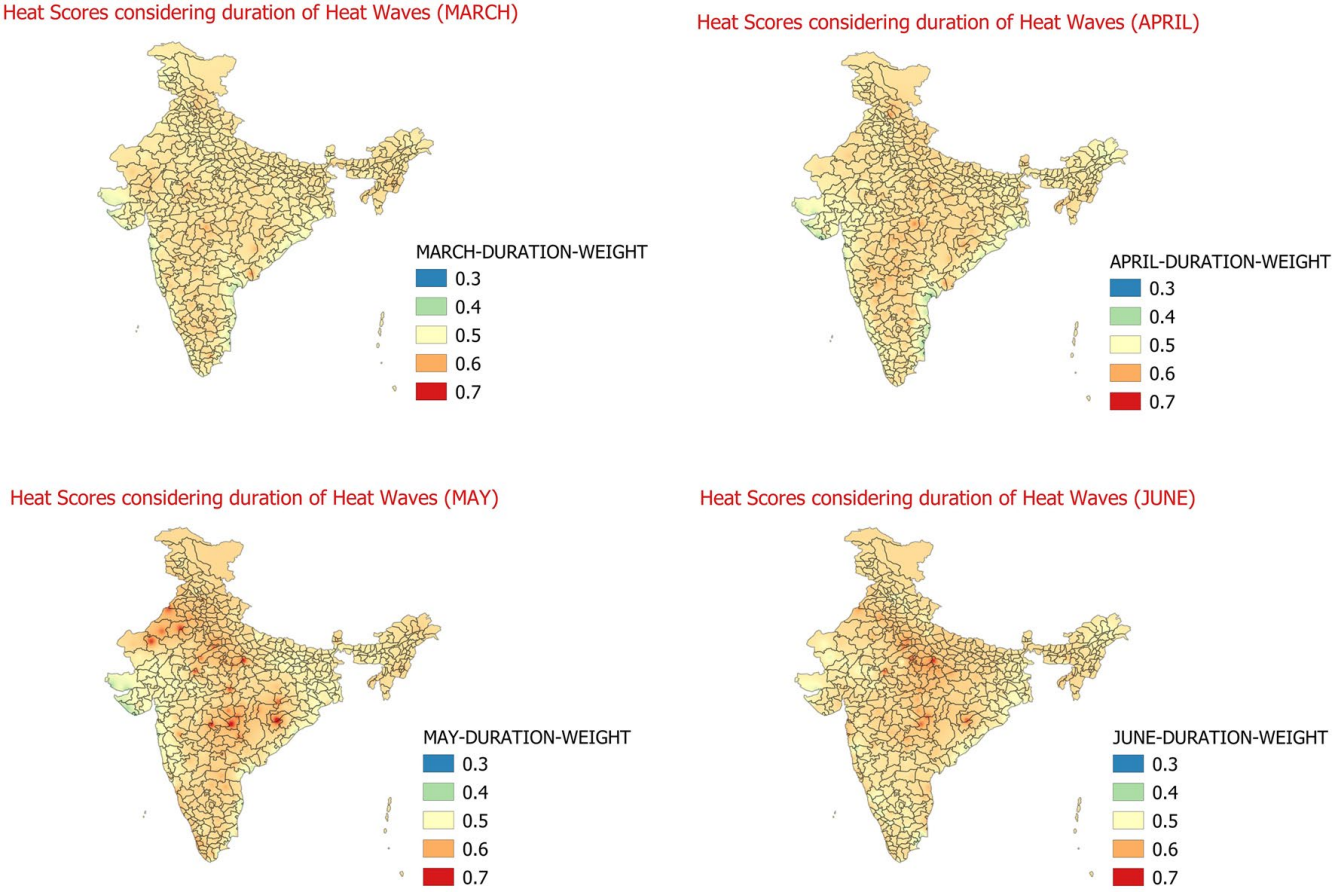
Daily average heat hazard scores considering only duration of heat wave/severe heat wave for the months of March (Top-left)﻿, April (Top-Right)﻿, May (Bottom-left)﻿, Jun (Bottom-right)﻿.

In the 5th step, an attempt is made to quantify the effect of duration/spell of heat wave days and severe heat wave days. for this purpose, each day of the month of March to June during 1981 to 2010 for each station is tagged as per its sequence in the heat wave or severe heat wave event. If the day under consideration is first day of heat wave/severe heat wave spell then the day is assigned the weight of 0; subsequently if the day is the 2nd consecutive day of any heat wave/severe heat wave spell, then it is assigned weight of 1, 3rd consecutive day of any heat wave/severe heat wave spell is assigned weight of 2 and if day is 4th consecutive day or more in any heat wave/severe heat wave spell, then the weight of 3 is assigned to that particular day. Based on this criteria, the weights are assigned to each day of the month (March to June) for the duration 1981–2010 and weight scores for all the days of a month are then aggregated and the daily average score for each month considering the effect of duration/spell of heat wave/severe heat wave is calculated. The daily average weight scores for each month related to duration of heat wave/severe heat Wave are shown below in Fig. 6. As the average scores are calculated on daily basis for a month the frequency of heat waves is inherently considered in the impact of heat waves/severe heat waves over any region and specifically no weights are considered for frequency of heat waves/severe heat wave events.

The Heat hazard scores of different meteorological variables analysed in this study are found to be independent of only Tmax based heat hazard scores. The dates of severe heat waves based on Tmax was extracted for random stations and for these dates corresponding scores for different analysed meteorological variables were calculated. These scores were found to be random and the severe heat wave dates based on Tmax doesn’t necessarily match the high scores of any of the analysed variables.

**Figure 7:**
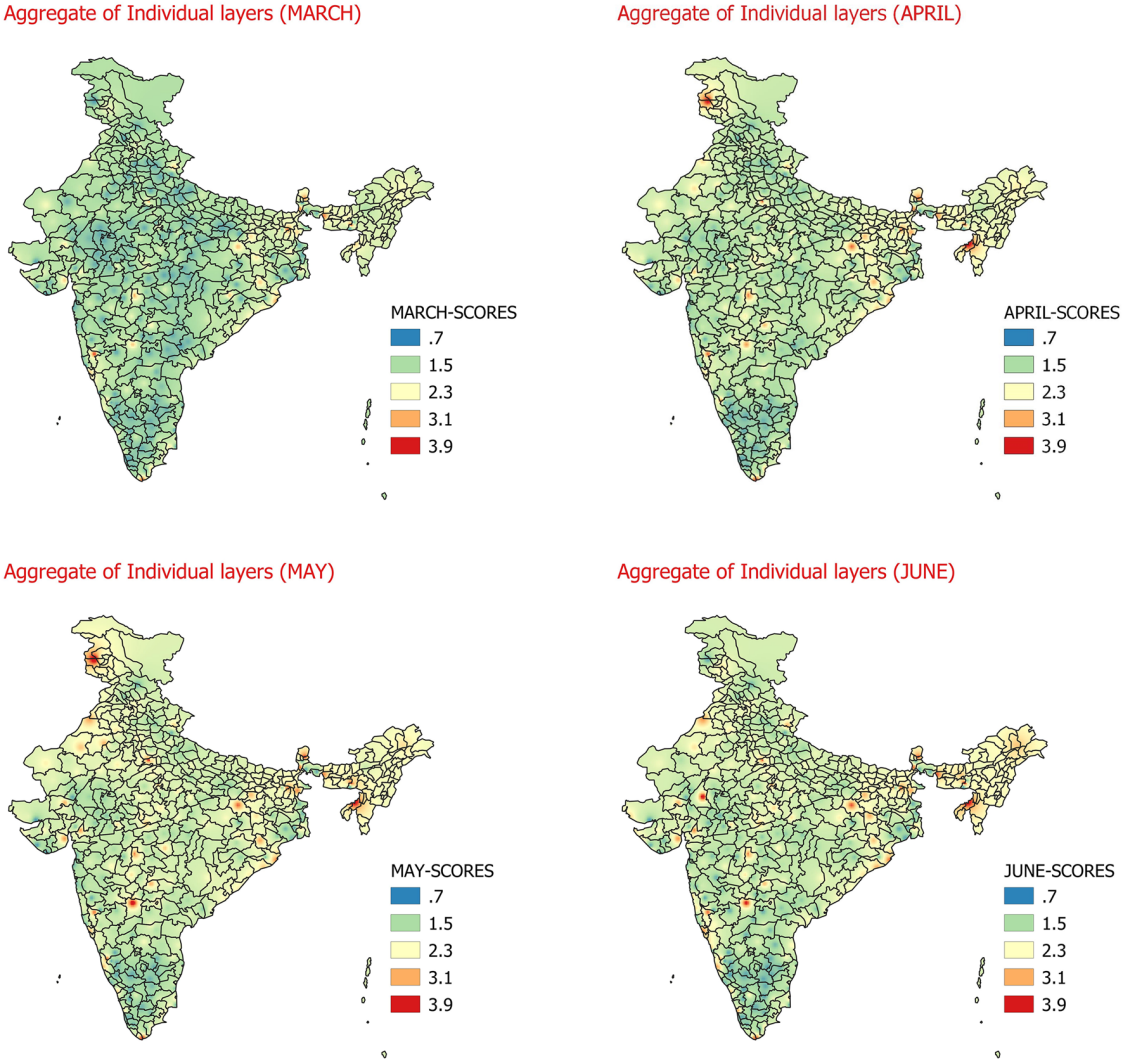
Daily average heat hazard scores considering maximum temperature, minimum temperature, relative humidity (09UTC), wind speed (09UTC), duration of heat waves March (Top-left)﻿, April  (Top-Right)﻿, May (Bottom-left)﻿, June (Bottom-right)﻿.

In the last step of the study, the heat hazard zonation of entire country is attempted by combining the scores which are calculated during 1st step to 5th step by considering different meteorological parameters. The number of stations considered for obtaining the weighted scores for meteorological parameters RH and WS (3rd and 4th step) are 158 while the number of stations for which temperature data is available and used in step 1st, 2nd, and 5th step is 300. As the number of stations differs for different meteorological parameters, therefore, to get the cumulative weight score from all the parameters considered in this study, we have utilized cell statistics functionality of Geographic Information System (QGIS)^40^. In this step all the weights from step 1 to step 5 are added to give the cumulative scores and are shown in Fig. 7.

These are the estimated final heat hazard weight score from this study which takes into account the effect of maximum temperature, minimum temperatures, relative humidity (09 UTC), wind speed (09 UTC) as well as duration of heat wave or severe heat wave spell.

## Discussion

The resultant Heat Hazard weight scores over India estimated from 1st to 5th step as described in the “Methodology and results” sections are divided into 5 parts to assign a danger level. Each of these part indicates the severity of danger associated with heat wave/severe heat wave events and are shown in Table 2.

**Table 2:** Danger Levels associated with different colour codes.

	Extremely high danger level of heat hazard
	Very high danger level of heat hazard
	High danger level of heat hazard
	Moderate danger level of heat hazard
	No or minor danger level of heat hazard

The Fig. 2 shows the heat hazard scores considering only Maximum temperatures. The Maximum temperature daily average weight scores for the months of May and June are higher than March and April pointing to the more impact of maximum temperatures in the month of May and June as compared to March and April. This study also suggests that during March and April month, some of the hilly regions of Himachal Pradesh and Jammu & Kashmir in the north and Arunachal Pradesh, Manipur and Mizoram in the north-east India are showing signatures of more impact of maximum temperatures. So these areas are prone to effect of heat related stress particularly due to maximum temperatures in the month of March and April. Apart from these regions, there are a few places in Rajasthan, Bundelkhand region of Uttar Pradesh and Madhya Pradesh, north Vidarbha and neighbouring regions of Madhya Pradesh, at a few places in interior Odisha and coastal Andhra Pradesh, where the impact of maximum temperature is found to be in very high danger level of heat hazard across April, May and June. The heat hazard scores based on Maximum temperatures are in good agreement with the monthly climatology of number of heat wave days from 1961 to 2010^9^. The heat hazard analysis as seen is in congruence with the monthly distribution of number of heat wave days which in turn is directly influenced by the movement of sun throughout the year^9^.

The Fig. 3 shows the heat hazard scores weight considering only minimum temperatures which are responsible to cause warm and very warm nights for the days when maximum temperatures were above normal. There is a visible movement of impact of minimum temperatures from south to north during the months from March to June. In the month of March the impact of minimum temperature is comparatively less than April to June months as minimum temperatures are largely on the lower side. The impact of minimum temperature looks to progress towards north in April month with central India and south peninsular India still being the centre of maximum impact on heat stress due to minimum temperatures. The impact weight scores due to minimum temperatures are seen over Vidarbha and neighbouring Marathawada, Rayalaseema, Telangana, interior Odisha, parts of Madhya Pradesh and at some pockets over Rajasthan and Uttar Pradesh. In the next month of May, the maximum impact of minimum temperatures over heat stress shifts towards north and covers almost entire central India and adjoining south peninsular India with maximum scores over Rajasthan and neighbouring Madhya Pradesh, southern parts of Uttar Pradesh, Delhi, Haryana and Punjab. In the month of June, the impact of minimum temperatures is largely concentrated over Rajasthan, Punjab, Haryana, Uttar Pradesh and Madhya Pradesh, however, the south peninsular India and adjoining parts of central India show significant decrease in the scores with respect to minimum temperatures. This is very much related to the reduced temperature in general due to the onset of southwest Monsoon whose normal date to cover south peninsular & central India is around 15th–20th June^30^. Therefore, the minimum temperatures have an important role in increasing the gravity of heat wave effect over different parts of the country in different months as presented above.

The Fig. 4 shows the weighted effect of relative humidity in increasing the impact of Heat Waves over the country. The presence of two seas on either sides of the Indian Peninsula and monsoon advancement from June onwards affect the distribution of moisture over the country^9^. In the month of March, the effect of RH is seen to aggravate impact of heat wave effect mainly over the Sub-Himalayan West Bengal, Gangetic West Bengal and adjoining areas of Odisha; at a few places over Konkan in Maharashtra, over southernmost parts of Tamilnadu and a few pockets over central & north India. The east and west coast gets the more relative humidity due to sea breeze in the afternoon whereas the northeast part of the country and north most part of the country see increase in RH due to Western Disturbances. In the month of April, the situation is similar to March with some areas over Jammu region and eastern parts of the country showing the increased effect of RH on heat waves severity. The April and May month also see the increased RH due weights due to the increased thunderstorm activities over east and northeast India leading to increase in the Relative humidity. The relative humidity is seen to have less impact on aggravating impact of heat waves over plains of northwest India and adjoining central India, which is very well correlated with the climatological lower relative humidity values over these regions^41,42^. In the month of June, again the situation is similar to March and April with additional few areas in Vidarbha, southern parts of Rajasthan and adjoining Gujarat showing more impact of RH over Heat Wave. This is due to the reason that the south-west monsoon covers approximately entire southern peninsular parts of India and some parts of adjoining central India by 15th–20th June bringing increase in the RH values. Therefore, the RH becomes an important criterion for regions of northeast India, Sub-Himalayan West Bengal, Gangetic West Bengal and adjoining areas of Odisha; a few places of Konkan in Maharashtra, southernmost parts of Tamilnadu and a few pockets of central & north India and Jammu mainly during the months of March, to June. However, during the month of May the share of RH in aggravating impact of Heat Waves is comparatively less over the plains of northwest India due to lower availability of RH.

The Fig. 5 shows the weighted scores for impact of surface wind speed on aggravation of heat waves. In the very high temperature regions, like in India where normal temperatures are more than 40 °C, the wind near surface is considerably hot (these winds are colloquially called as “LOO” in some parts of India) which leads to increase in the body temperature due to passing over of the high-temperature winds, especially over north and central India. This can be understood as effect opposite of wind chill during winters. In the coastal regions (east coast and west coast of India), the impact of heat waves largely depends on the strength of land breeze and sea breeze and their onset and duration. Therefore the wind speed is considered to understand these effects over coastal and interior regions of India during March to June when surface temperatures are very high for most parts of the country. The high wind speed is generally associated with the thunderstorm activities in the pre-monsoon and hot weather season of the country. There have been number of studies on climatology and the areas where we find more impact of wind speed on heat waves are in congruence with these studies. Climatologically it is found that in March maximum thunderstorm activity are located at three corners of the country i.e., Assam, Kerala and western Himalayas^43^. During April and May, chief areas observing thunderstorms are Assam, Meghalaya and Sub Himalayan West Bengal in the northeast and Kerala and adjoining Tamil Nadu in the south and Jammu & Kashmir in the north^43^. There is marked change in the thunderstorm activity during June, whereby there is increase in thunderstorm activity over central and western parts and northern plains associated with the advance of south-west monsoon and, there is reduction in the thunderstorm seen over southern parts of the country^43^. Similar to the results from the climatology of thunderstorms, in this study it is found that in all four months of March, April, May and June the share of wind speed on heat wave impact is mostly observed over Sub-Himalayan West Bengal, Gangetic West Bengal and adjoining regions of north east and east India and over some regions in south peninsular India mainly over Vidarbha and adjoining Marathwada, few areas of Konkan and southernmost parts of Tamilnadu. The aggravating effect of wind speed over Heat waves is also seen over Jammu region during April and May and over Gujarat, Saurashtra and adjoining areas during months of May and June. Most of the plains of northwest India and parts of central India are found to have less impact of wind speed in aggravating effect of heat waves which are also similar to regions experiencing lesser frequency of thunderstorms.

The Fig. 6 shows the weighted scores of the impact of heat wave spell duration in aggravating the impact of Heat Waves. Climatological studies have found that May and June are the months where average number of HW days experienced and spatial coverage of HWs were relatively more than during March and April^9^ with many areas from northwest India, eastern parts of Peninsula and some areas of central India recording more than ≥ 4 HW days during these two months. Among May and June also the May month experiences more number of average heat wave days with more spatial coverage particularly over the regions of west Rajasthan, Odisha, Vidarbha, Telangana and coastal Andhra Pradesh^9^. Similarly, it was found that the severe heat Wave days are on average found only during the months of May and June over northwest, north and eastern parts of the country^9^. Similar, to these findings, the duration of heat wave event is seen to aggravate the impact of heat waves mostly during May and June months on the same regions. During March and April the duration of heat wave events plays less significant role in aggravating impact of heat waves. During March and April, the heat wave spell/duration seems to have some impact at a few places over Himachal Pradesh, East Madhya Pradesh, Vidarbha, southern parts of interior Odisha and coastal Andhra Pradesh and southern parts of Tamilnadu. In the April month, the heat wave spell/duration is seen to have some role in aggravating the impacts of Heat Waves over south peninsular India including Vidarbha, Chhattisgarh, Telangana, Rayalaseema, and interior Odisha, coastal Andhra Pradesh, Marathawada, Madhya Maharashtra, interior Karnataka and northern parts of Tamilnadu. Other than these regions, some areas of southeast Uttar Pradesh and Himachal Pradesh also experiences aggravated heat waves impact due to longer duration/spell of Heat Waves. Major impact of duration of heat waves is seen during months of May and June. The share of impact of duration of heat Waves majorly is seen over parts of Rajasthan, Madhya Pradesh and adjoining Uttar Pradesh and Vidarbha and adjoining Chhattisgarh and Odisha. The heat wave spell therefore, becomes more important over northern parts of southern peninsular India and adjoining areas of east India and over central and north/northwest India during May and June in aggravating the impact of heat waves.

The Fig. 7 shows the aggregate impact of all the parameters described in Figs. 2, 3, 4, 5 and 6 (Step 1st to 6th). The maximum spatial coverage of extremely danger and maximum danger hazard scores with respect to heat waves utilizing associated meteorological parameters and duration of heat waves is seen during the months of May, followed by June, April and March respectively. It can be inferred from Fig. 7 that in the month of March, the cumulative Heat hazard score is maximum over East India which includes, Bihar, Jharkhand, West Bengal, Parts of Odisha and parts of Northeast India. Apart from these regions, aggravated heat wave impact is also observed over northern parts of south peninsular India and some parts over southern parts of Madhya Maharashtra and southern most areas of Tamilnadu. In the month of April the regions with aggravated impact of heat waves are same as that of the regions seen in March but with more weighted scores signifying more impact of heat waves in April as compared to March. In addition to the regions observed in the month of March, Jammu region and some areas of west Rajasthan and Gujarat are found to have aggravated impact of heat waves. In the month of May, some regions around Gujarat/Saurashtra and Punjab, Haryana and neighbouring Rajasthan, parts of central India, and east and west coast also observes increased aggravating impact of Heat Waves in addition to regions observed during month of April. The Heat Hazard scores are found to be maximum with maximum spatial coverage during the month of May. From the Fig. 7, it can be inferred that in the month of June, except for Jammu region, the situation spatially is similar to what was observed in month of May. The heat hazard scores however, are on the lower side as compared to month of May particularly over the southern and western parts of the country.

These weighted scores as derived identify the regions in the country for which impact of heat wave events could be more devastating considering the different meteorological parameters including maximum temperatures, minimum temperatures, relative humidity, wind speed and duration of heat wave event. Out of the studied meteorological parameters and duration of heat waves, some of the parameters may significantly influence Heat Wave impact over the region of interest while other parameters may not as already discussed with help of individual weighted scores presented in Figs. 2, 3, 4, 5 and 6. Therefore, there is flexibility for the forecaster’s to identify the parameters important for their region of interest and use these scores as threshold values to issue impact based Heat Hazard warnings. For this purpose we have supplied the weighted scores with different permutations and combinations of meteorological parameters and duration of the heat waves in the supplementary text (Figs. S1–S4). Forecaster’s by assigning the weights as discussed in this study to the different identified forecasted meteorological parameters can calculate daily Heat hazard scores. The regions with higher scores relative to the findings in this study are probable areas to face aggravated impact of Heat Wave related threats. It is also seen from the study that the regions of high scores based on variables other than Tmax are small and scattered.

**Figure 8:**
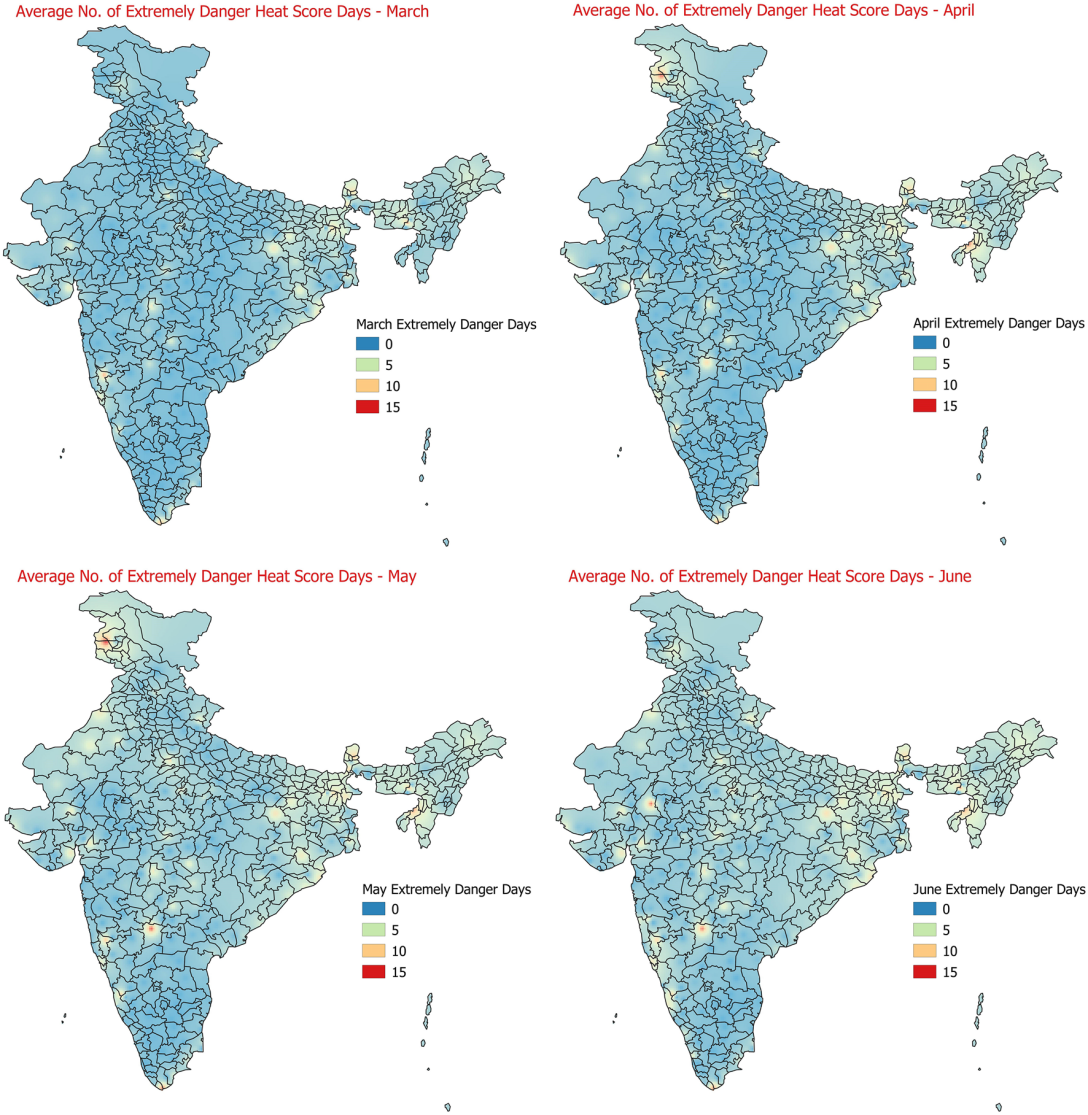
Average number of days in the months of March to June under the category of extremely danger level of heat hazard.

**Figure 9:**
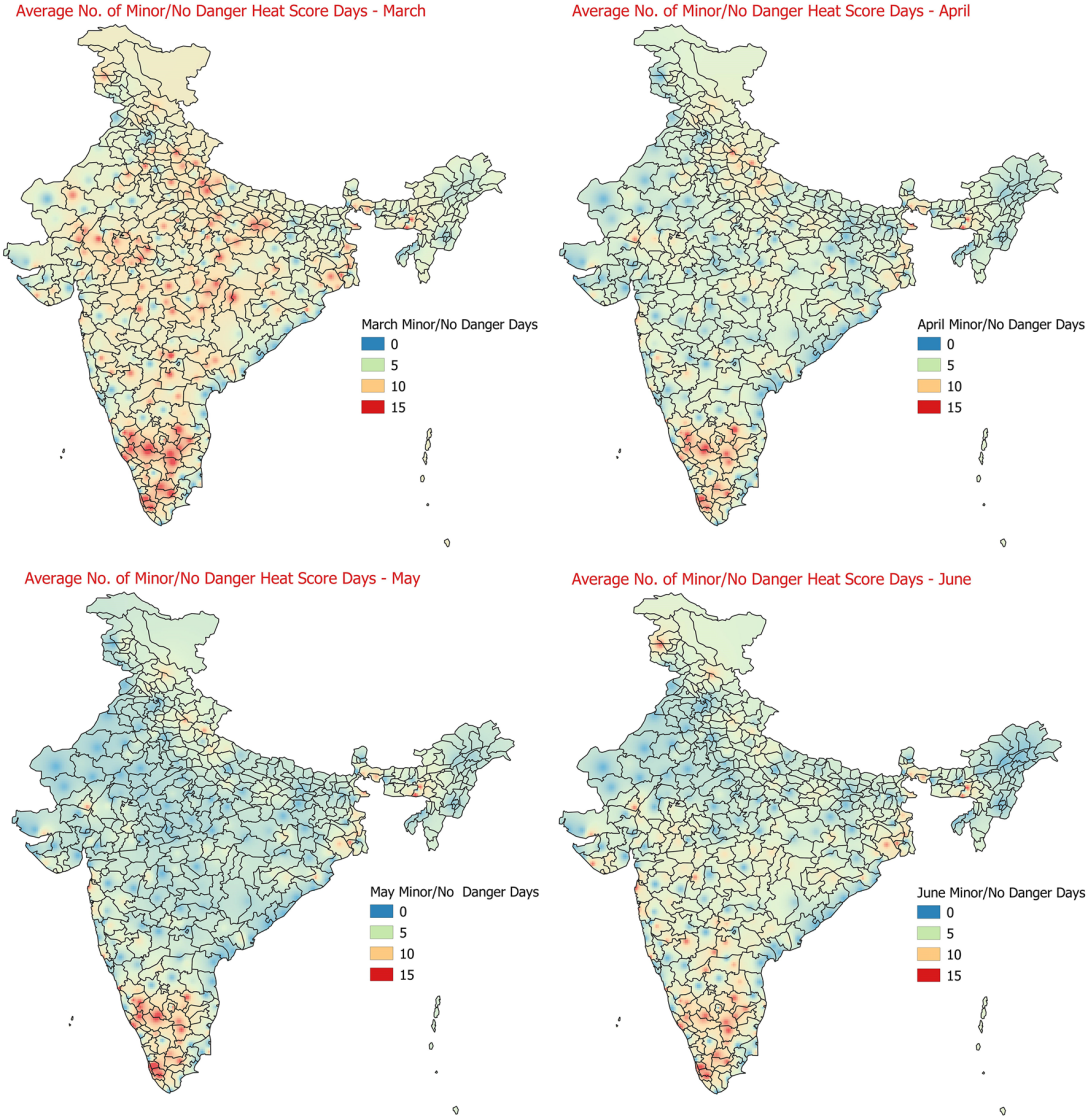
Average number of days in the months of March to June under the category of minor/no danger level of heat hazard.

The region wise average number of days under different categories of Heat Hazard scores as described in Table 2 for each of the months of March to June were also calculated. The Figs. 8 and 9 below shows the average number of days in the months of March to June under the category of extremely high danger level of heat hazard and minor/no danger level of heat hazard scores. May months observes maximum average number of days in extremely high danger level of heat hazard, followed by June. March observes minimum number of days with extremely high danger level of heat hazard scores. The average number of minor/no danger heat score days shows that their numbers are maximum in the extreme parts of southern peninsular India (Kerala, Tamilnadu and Karnataka) during March to June making them less prone to heat hazards in general. For other categories of heat hazard scores as per Table 2, the monthwise average number of days are provided in the supplementary text (Figs S5–S7).

## Conclusion

Heat Wave impact gets aggravated by variety of meteorological as well as socio-economical parameters. Efforts are going on to explore the full array of factors that drive heat stress, including possible synergistic effects of demography, society, economies, pollution etc. which may vary from place to place. Also the factors which are understood to be affecting heat wave events are also changing with time, making it more difficult to have a universal definition and thresholds to assess the impact of Heat Wave events. In this study an attempt is made to quantify the impact of different meteorological parameters in aggravating heat wave impacts and thereby leading to heat wave hazard zonation of the country. In nutshell, the cumulative heat wave hazard impact is inferred to be maximum over Bihar, Jharkhand, West Bengal, Parts of Odisha and parts of Northeast India, over some parts of Bundelkhand region and neighbourhood, over northern parts of south peninsular India and some parts over southern parts of Madhya Maharashtra and southern most areas of Tamilnadu during the months of March to June and also over Jammu region during April and May and over Gujarat and Saurashtra region during May and June.

This study also points out the relative contribution of different meteorological parameters having differential share in aggravating the impact of heat waves over different regions of the country during different hot weather months. Thereby, providing an opportunity for the weather forecasters and disaster managers to focus on these parameters for effective location/month specific impact based early warning services and mitigation initiatives. This study is an attempt to provide an effective trigger approach which could be utilized by weather forecasters to issue impact based heat wave related warnings which could be utilized by public health and disaster managers for taking effective heat stress mitigation initiatives. As the underlying factors keep changing these trigger points needs to be periodically re-evaluated based on the developed scientific understanding and by verification with the data.

## Supplementary Information

Supplementary Figures.

## Data Availability

The datasets generated during the current study are available from the corresponding author on reasonable request. The datasets analysed during the current study are available with the National data Centre, India Meteorological Department, Pune and can be accessed through the data supply portal https://dsp.imdpune.gov.in/.
