# Supplementary material for: Hot weather hazard analysis over India
Source: Sci Rep. 2022 Nov 17;12:19768. doi: 10.1038/s41598-022-24065-0 (PMC9671900; doi:10.1038/s41598-022-24065-0)
Supplement: Supplementary file 1 — Supplementary Figures. [file 41598_2022_24065_MOESM1_ESM.docx]

**Hot Weather Hazard Analysis over India**

Akhil Srivastava, M. Mohapatra, Naresh Kumar

India Meteorological Department, Ministry of Earth Sciences,

Government of India.

Email: - akhils.imd@gmail.com

| 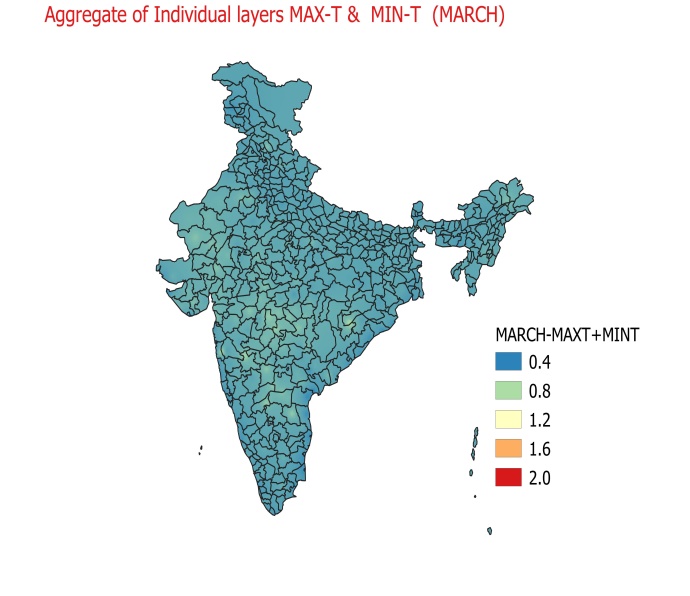 | 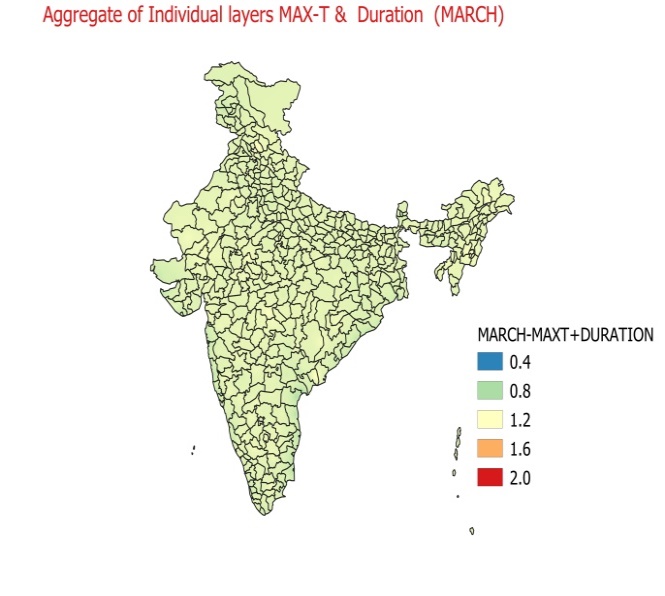 |
| --- | --- |
| 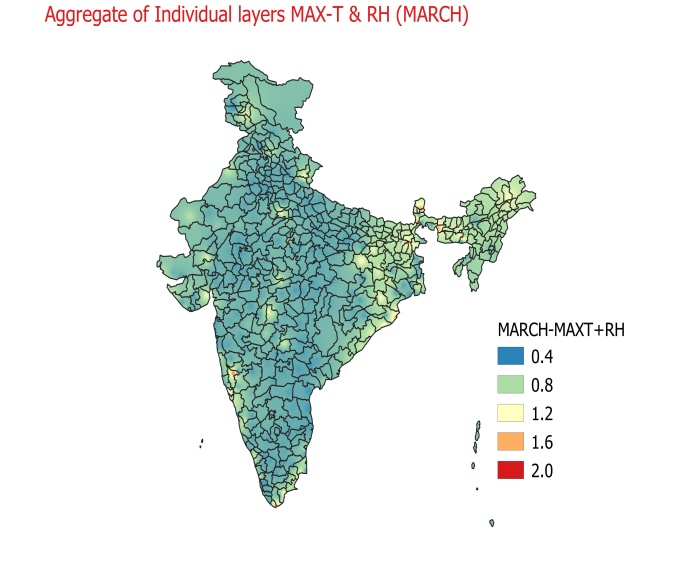 | 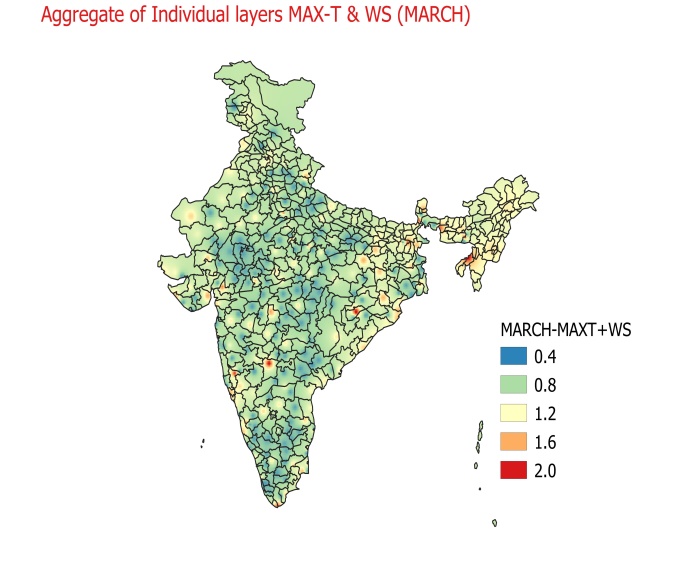 |
| 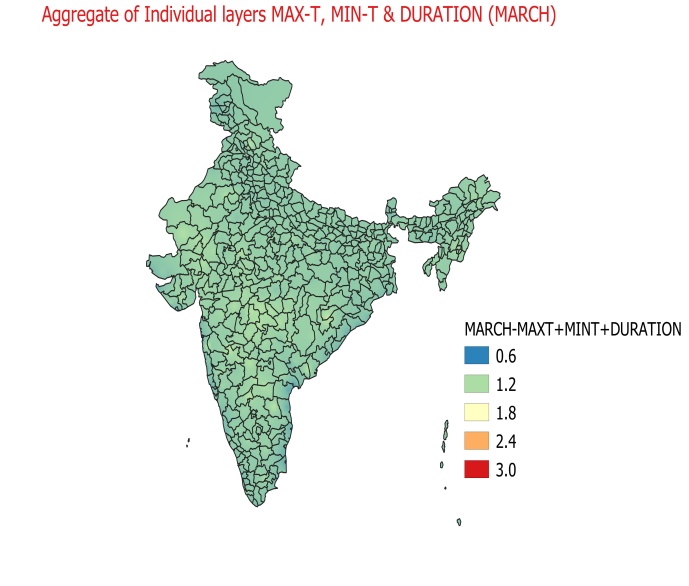 | 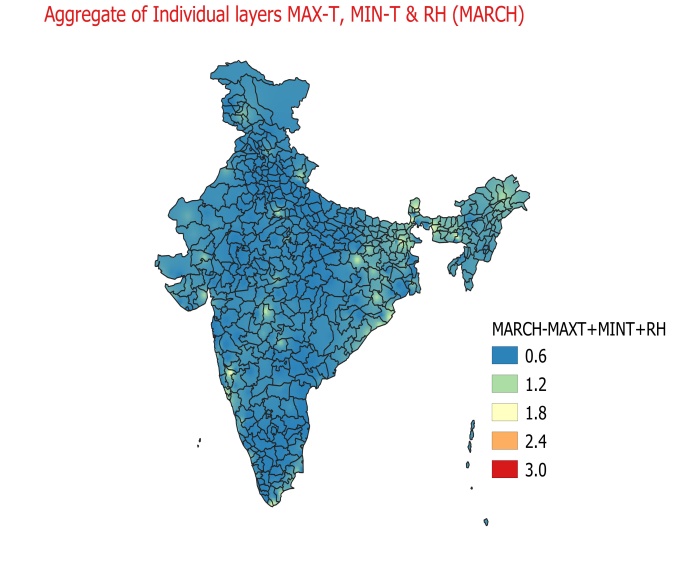 |
| 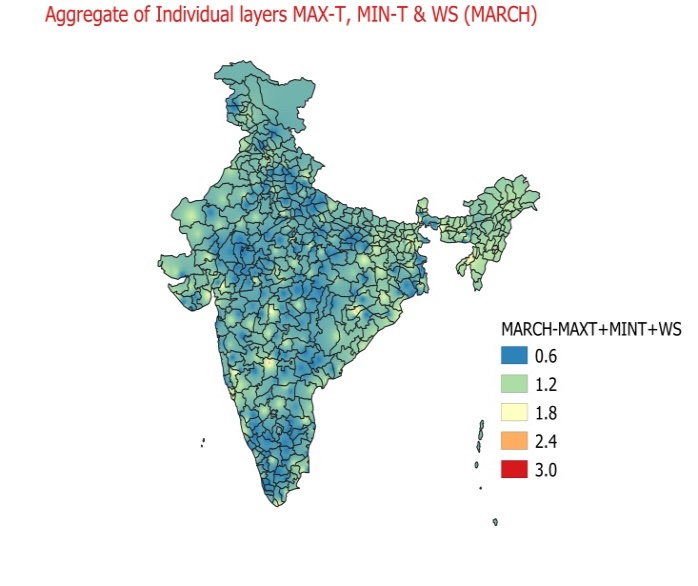 | 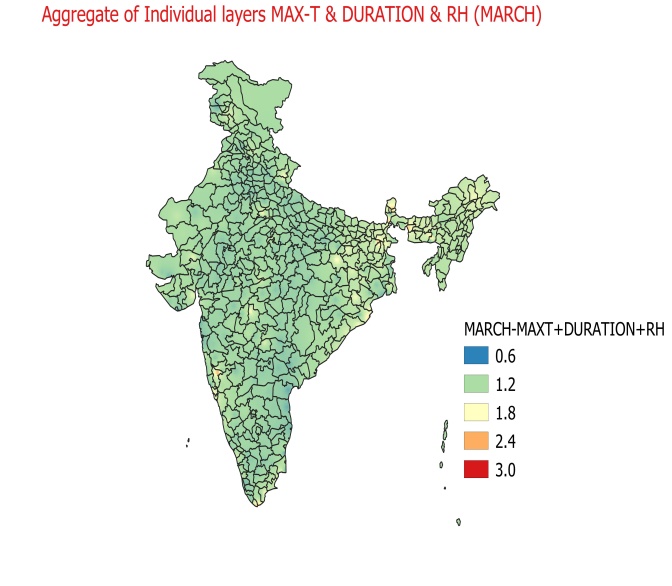 |
| 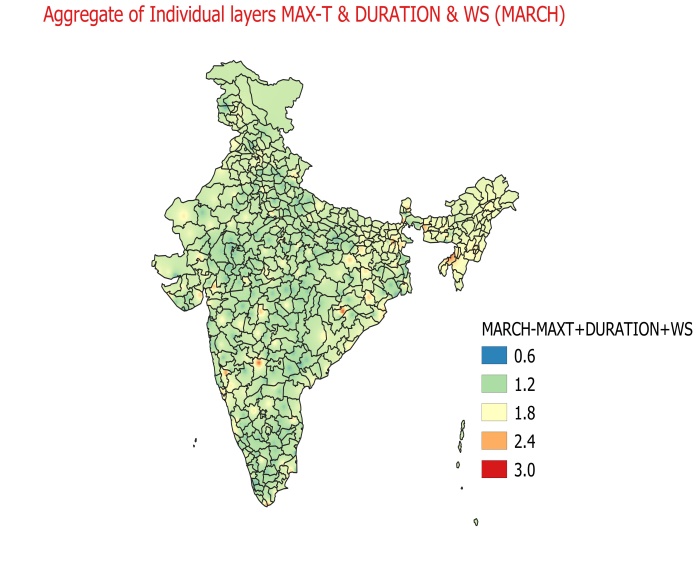 | 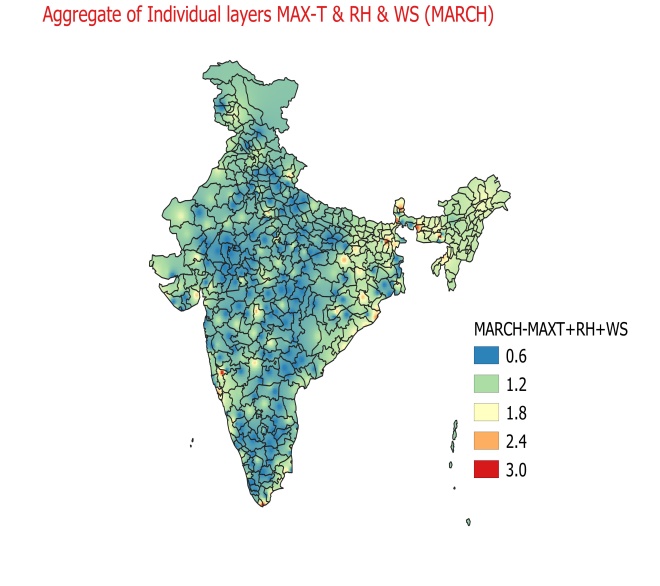 |
| 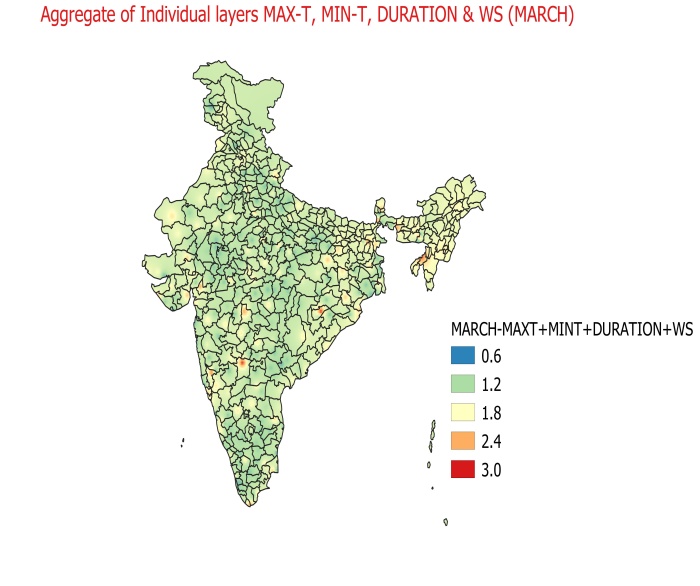 | 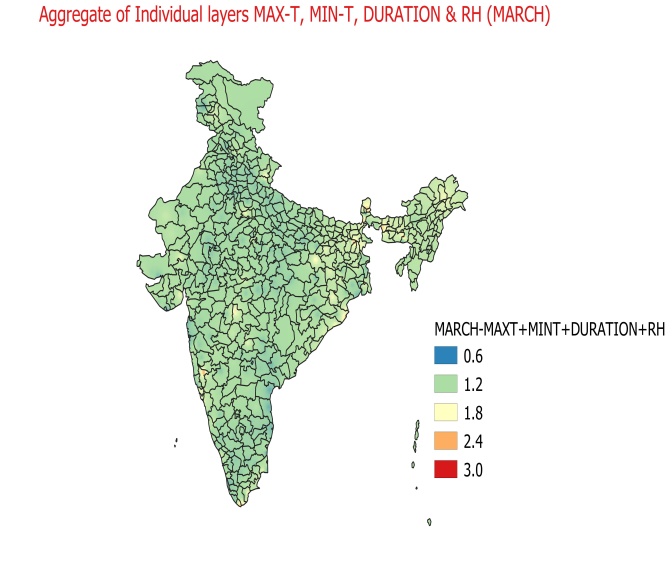 |
| Fig S1. March month mean daily weighted scores by aggregating different permutation and combination of meteorological parameters having influence on heat waves impact. | |

| 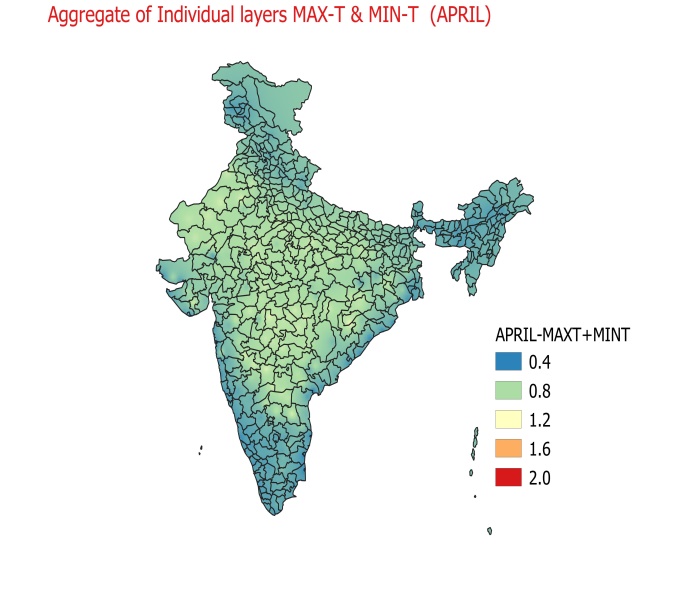 | 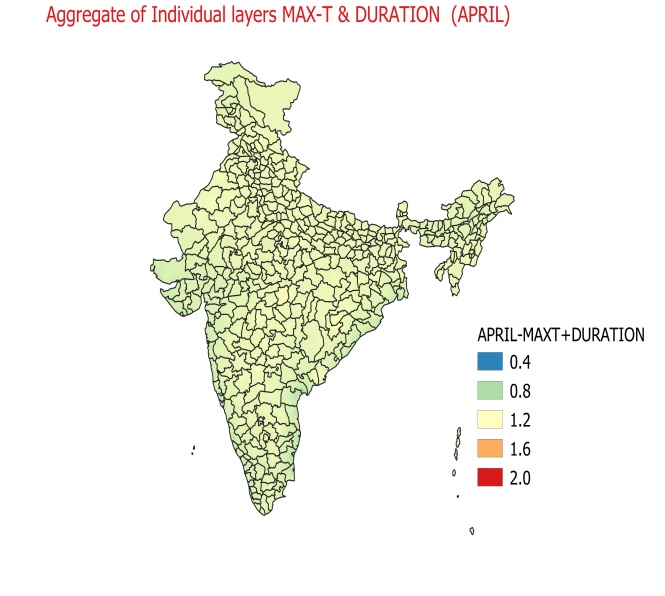 |
| --- | --- |
| 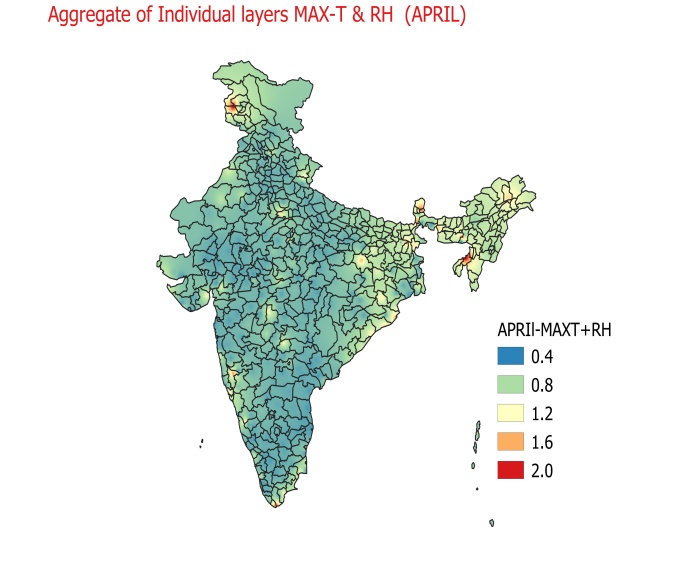 | 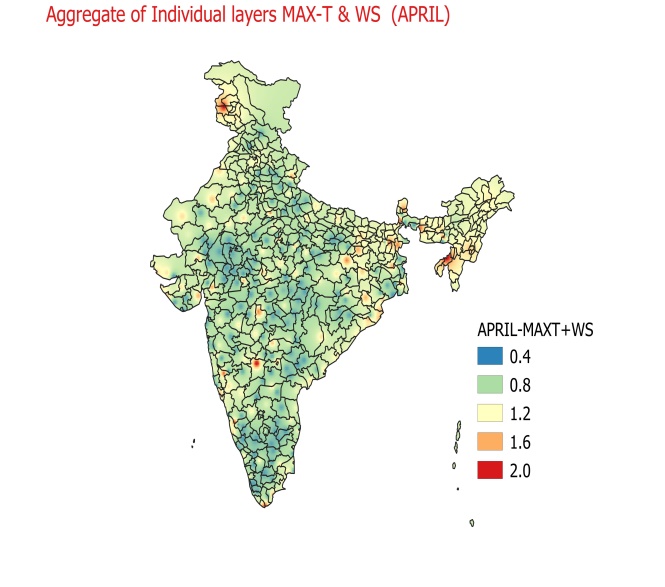 |
| 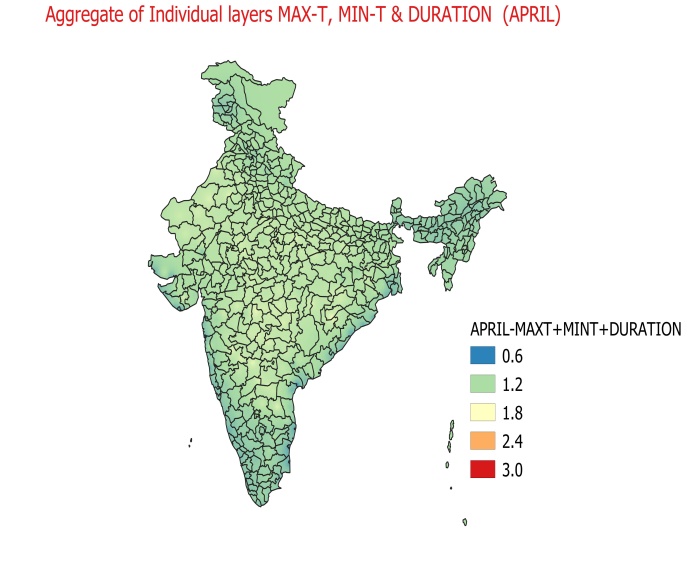 | 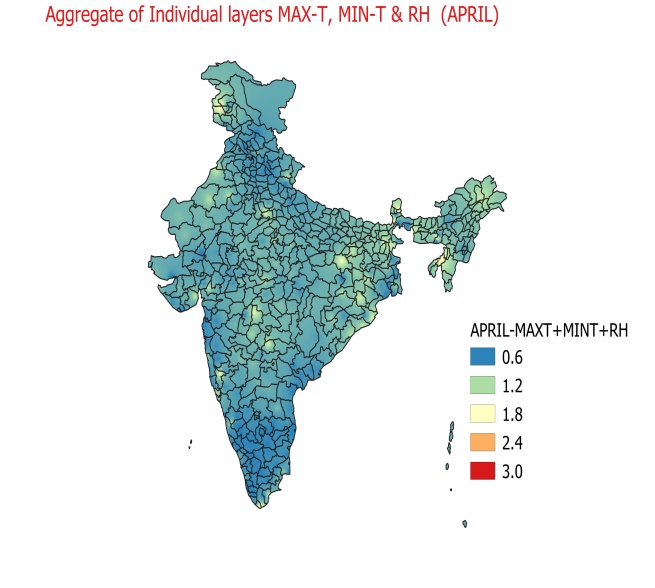 |
| 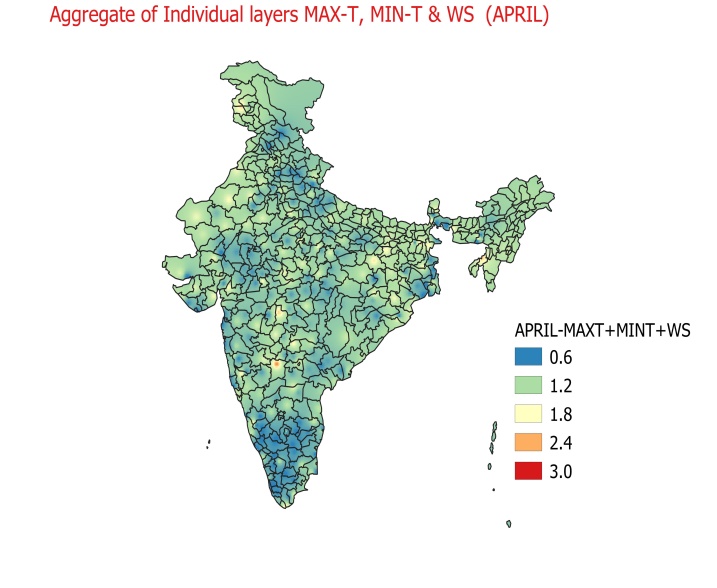 | 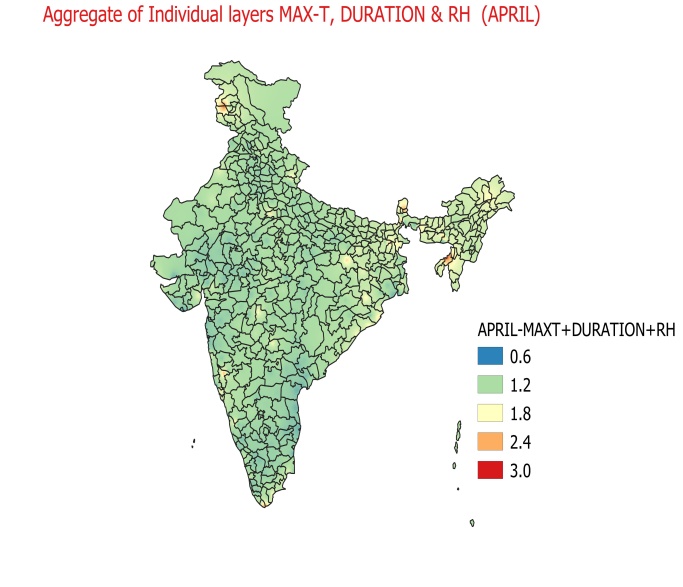 |
| 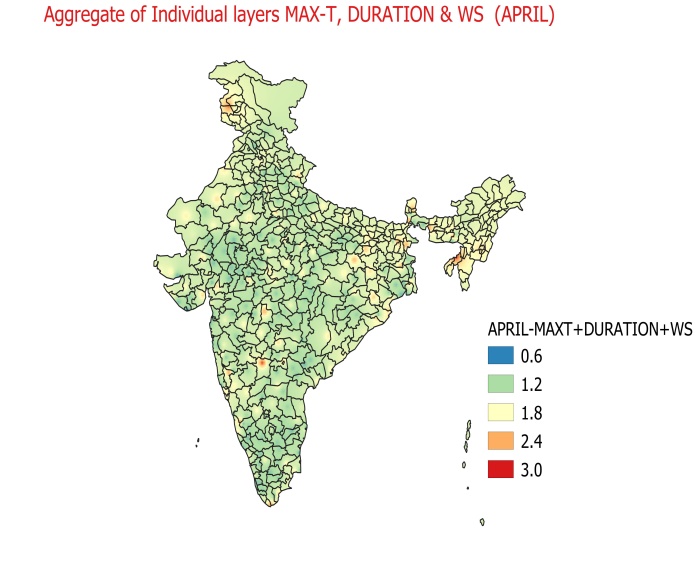 | 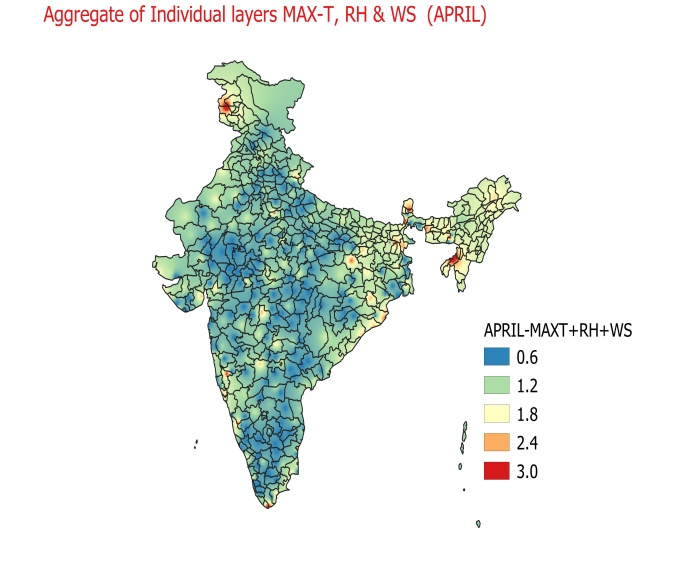 |
| 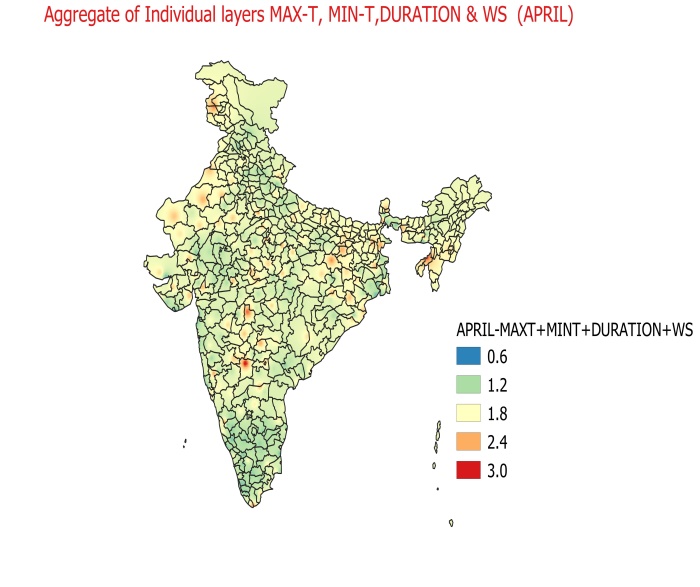 | 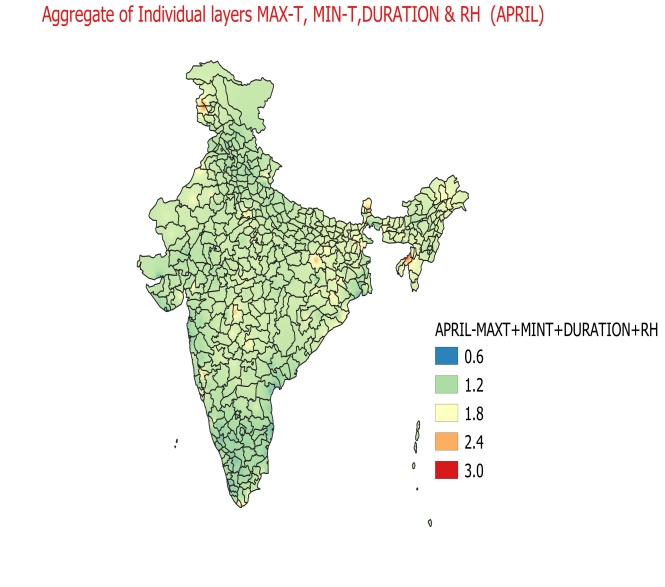 |
| Fig S2. April month mean daily weighted scores by aggregating different permutation and combination of meteorological parameters having influence on heat waves impact. | |

| 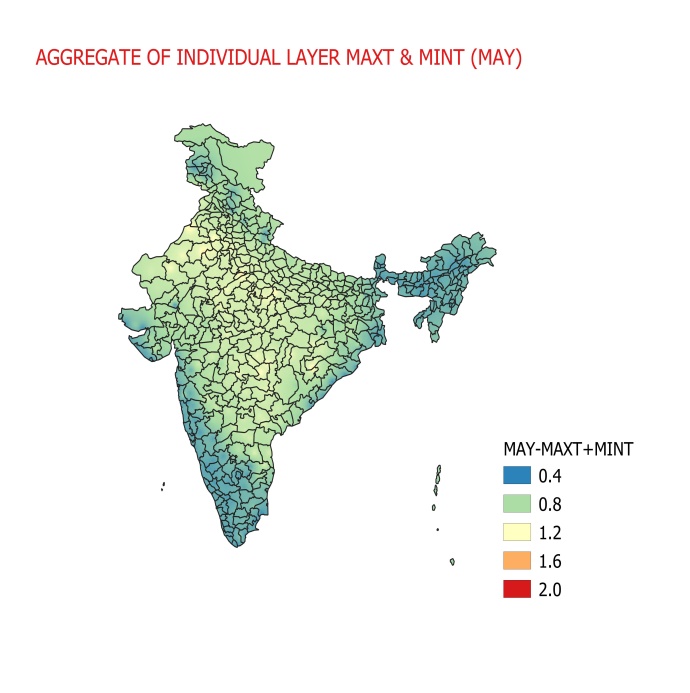 | 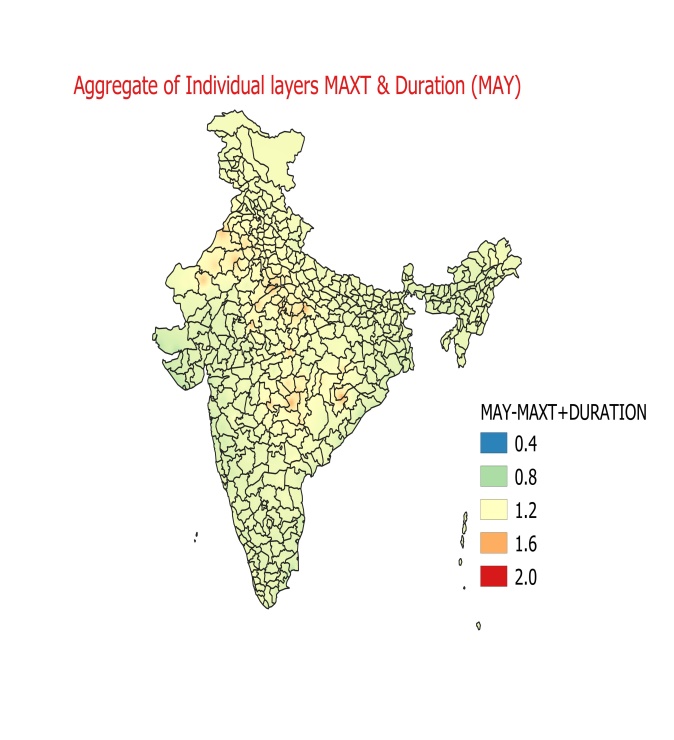 |
| --- | --- |
| 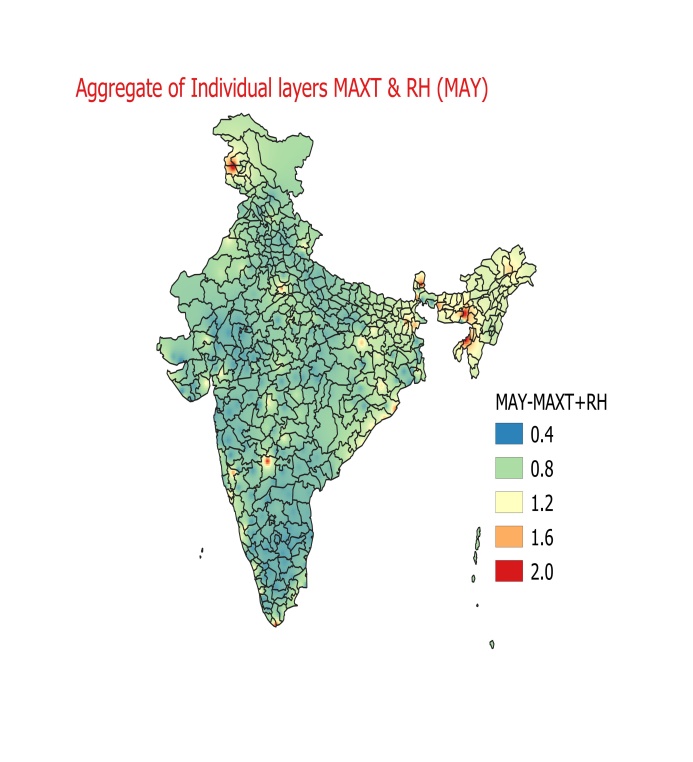 | 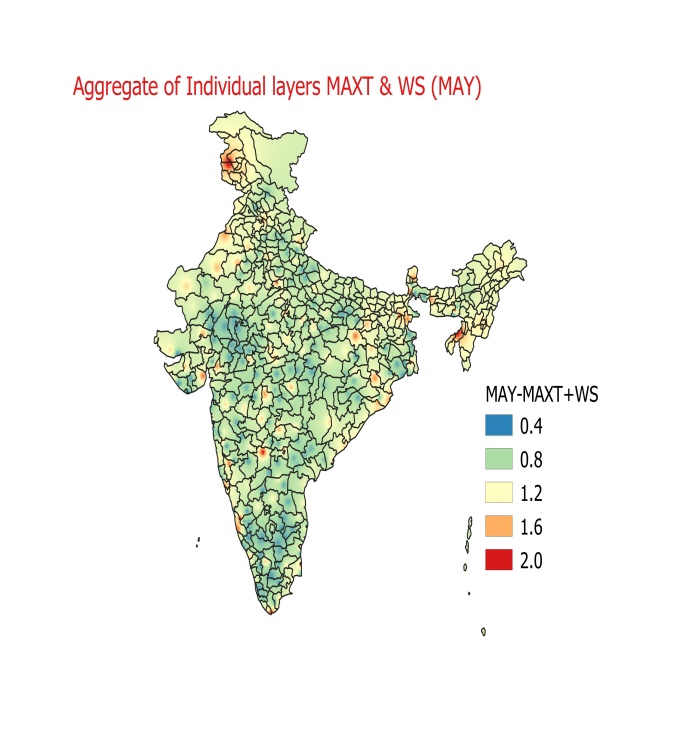 |
| 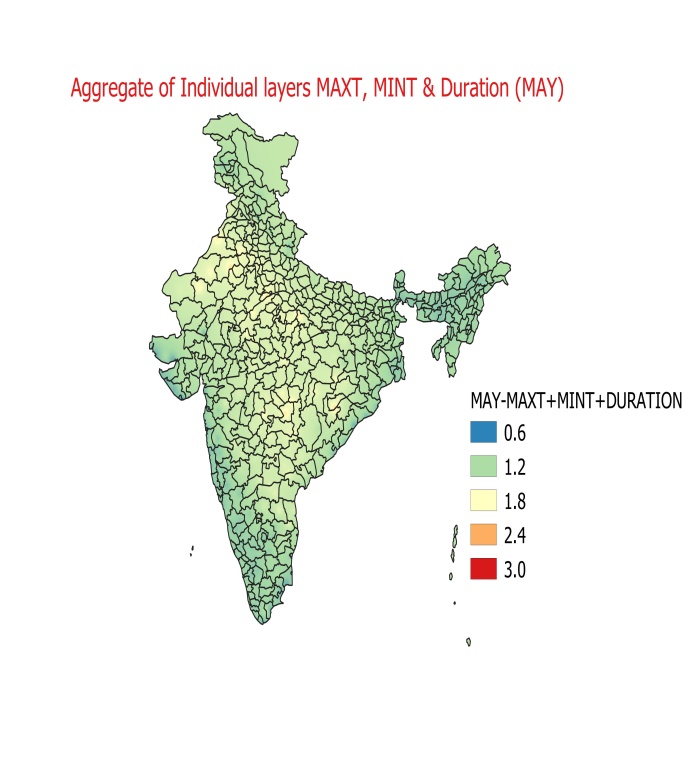 | 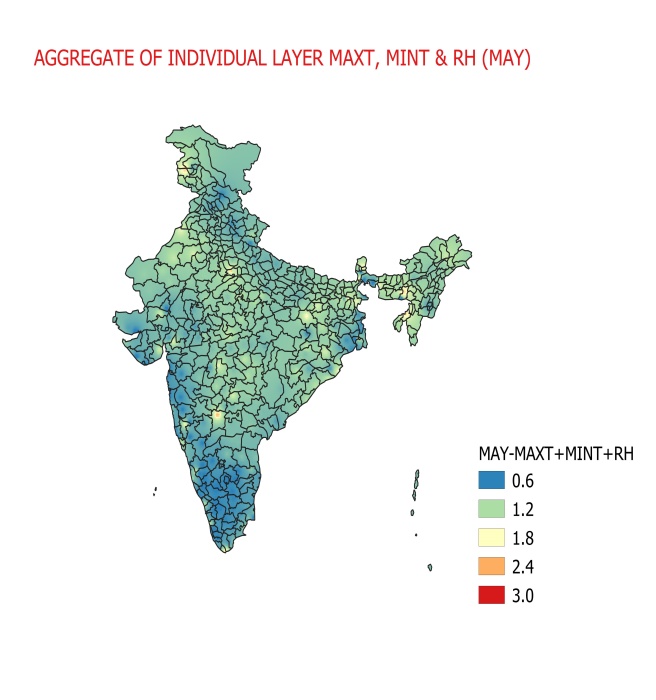 |
| 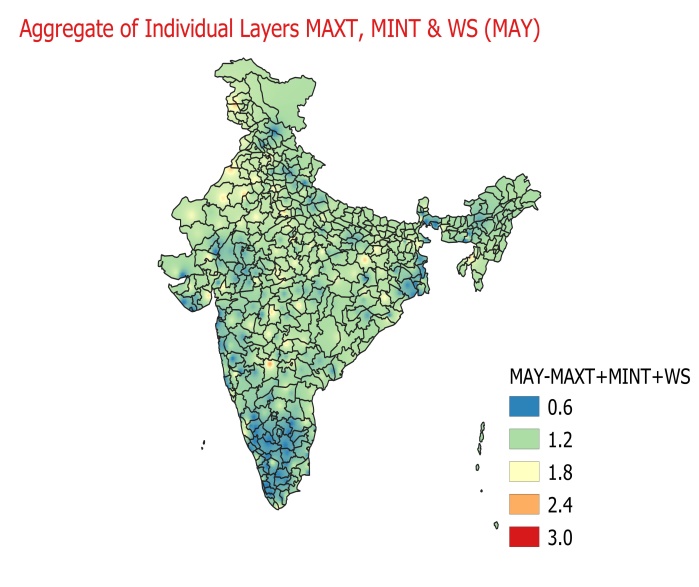 | 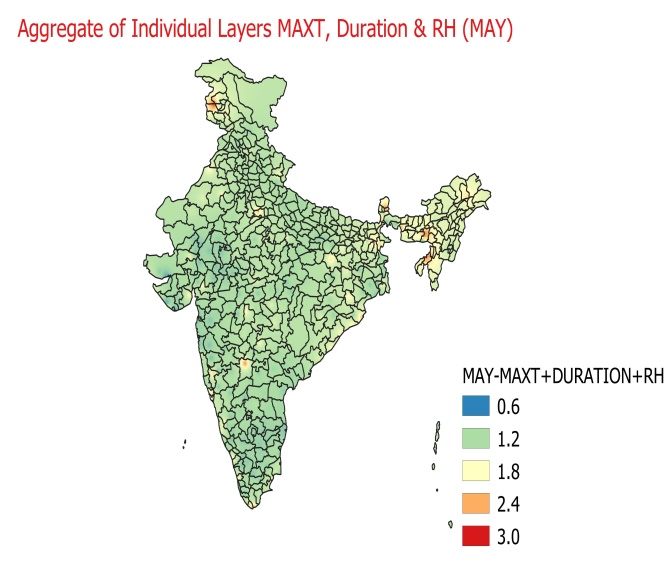 |
| 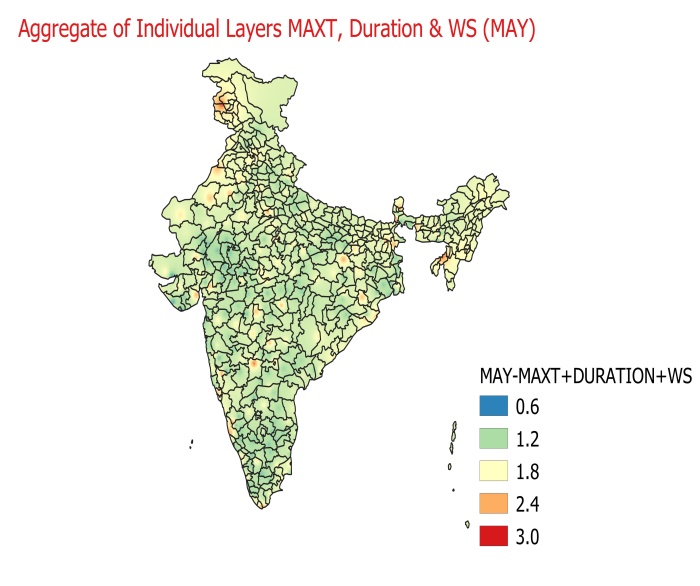 | 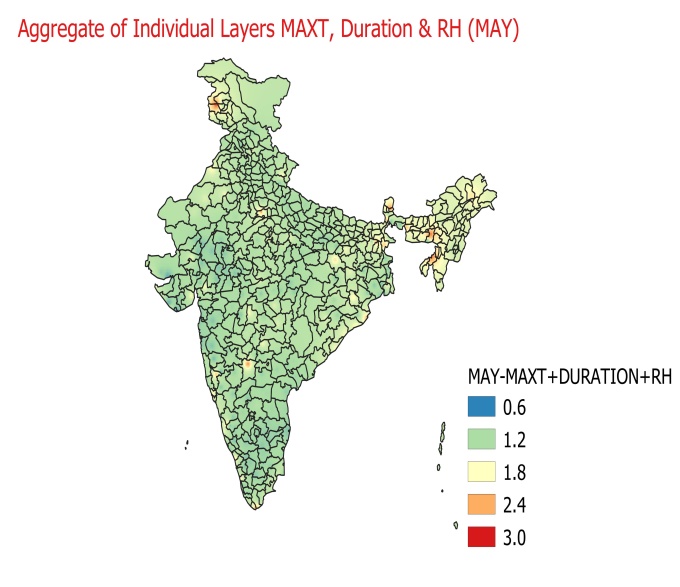 |
| 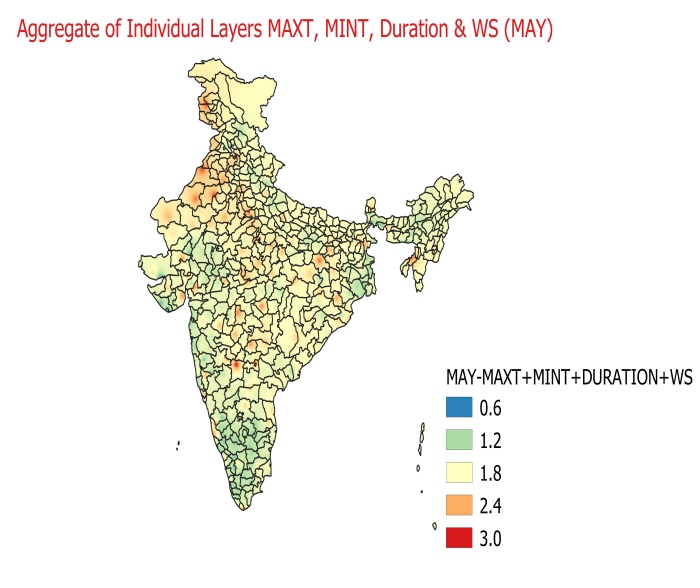 | 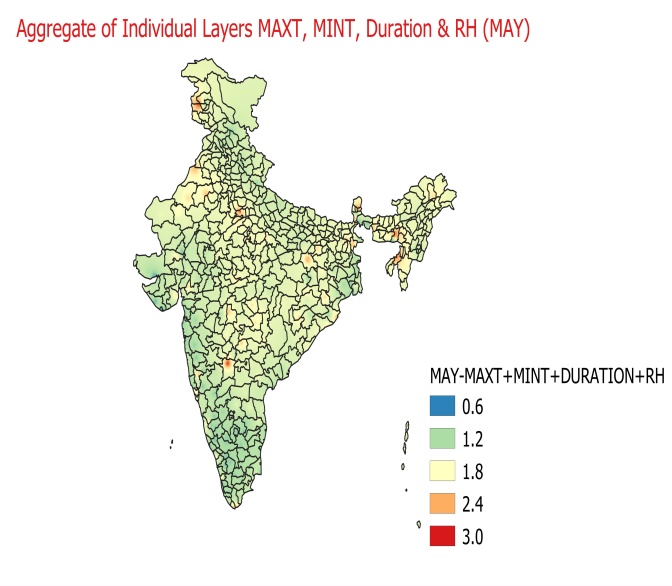 |
| Fig S3. May month mean daily weighted scores by aggregating different permutation and combination of meteorological parameters having influence on heat waves impact. | |

| 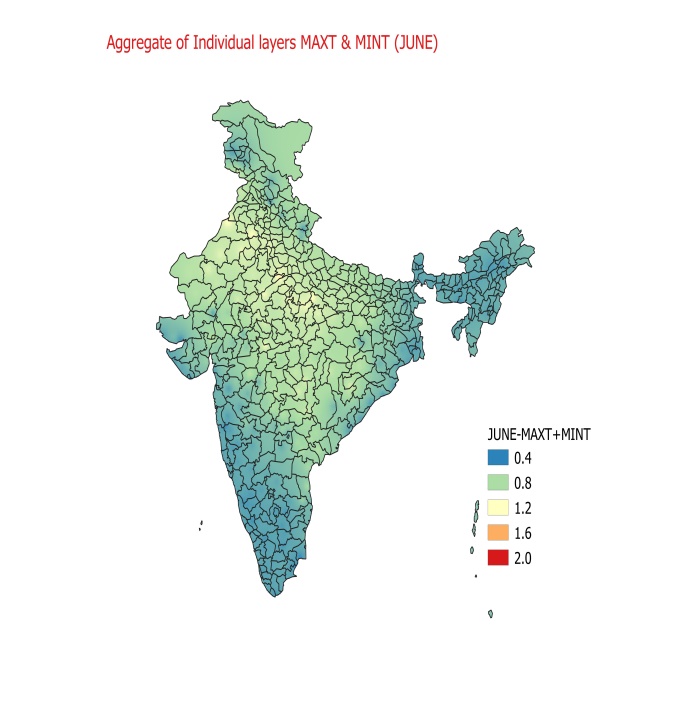 | 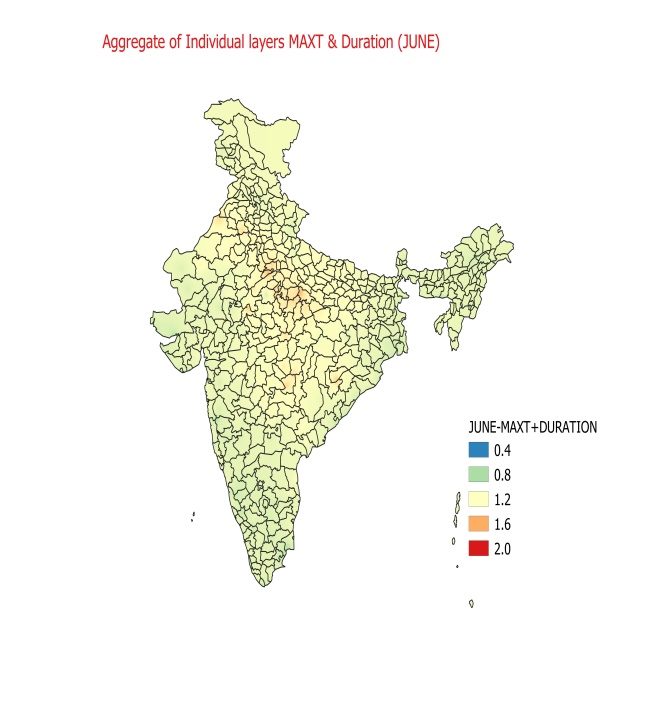 |
| --- | --- |
| 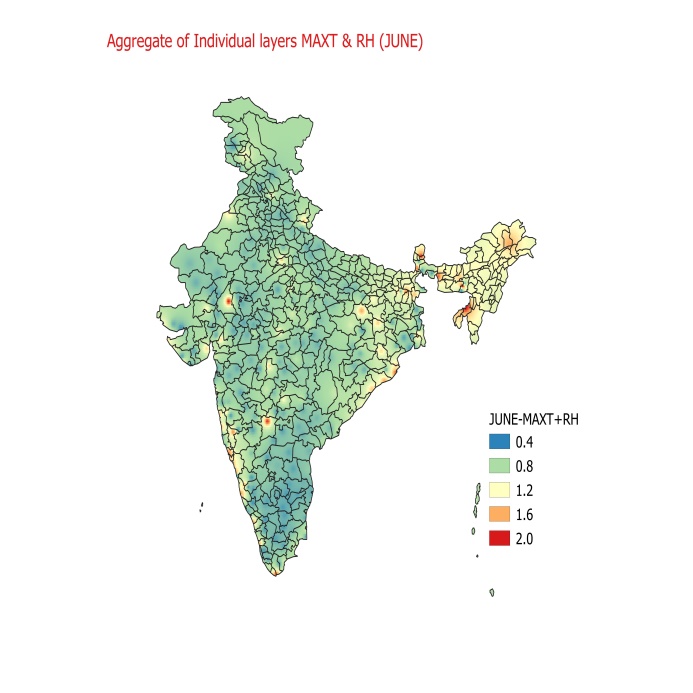 | 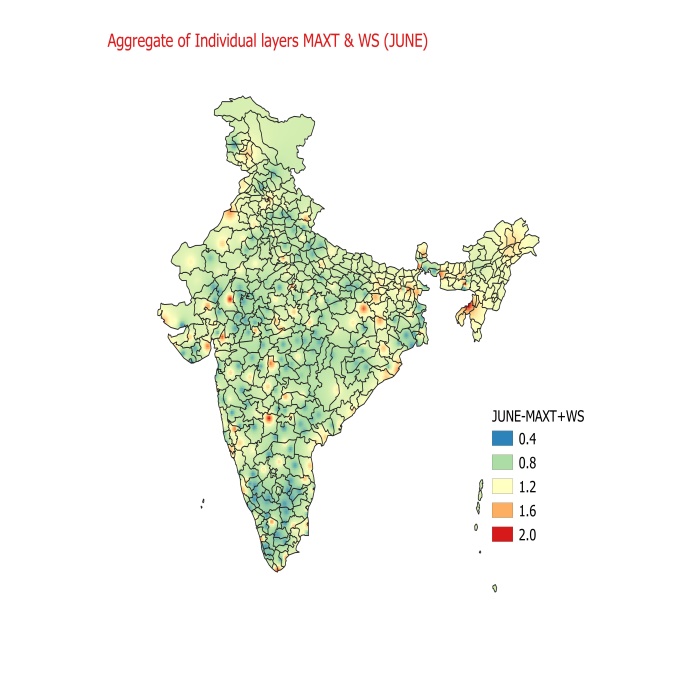 |
| 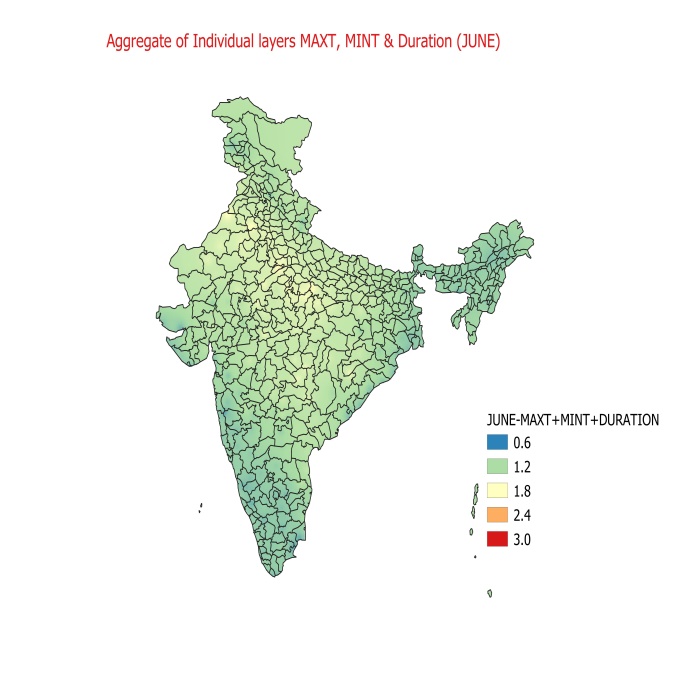 | 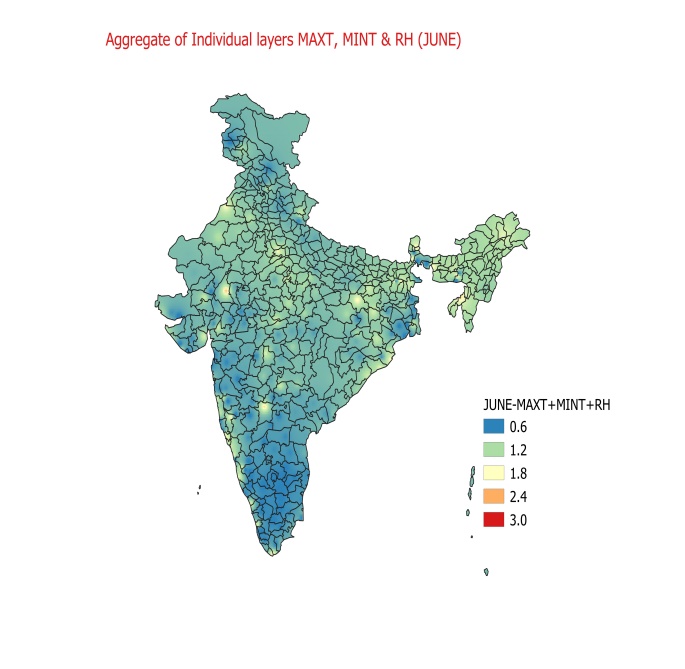 |
| 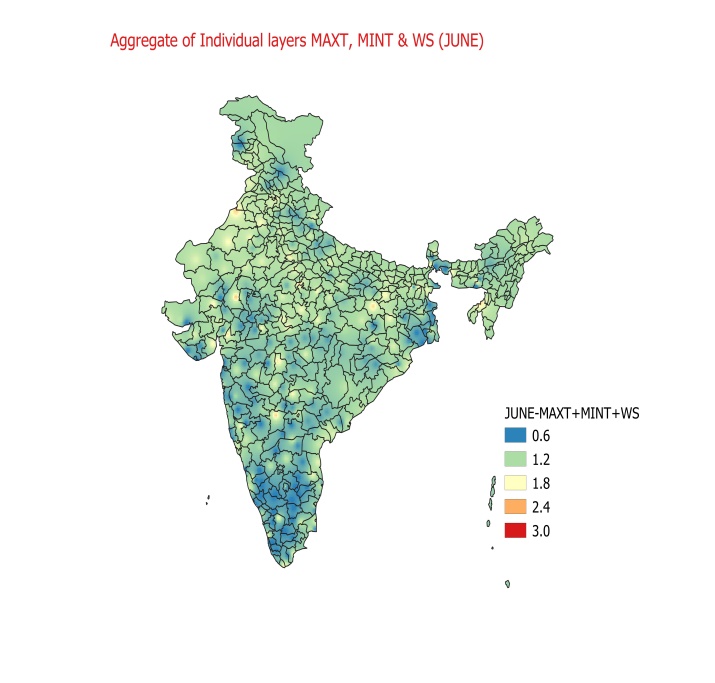 | 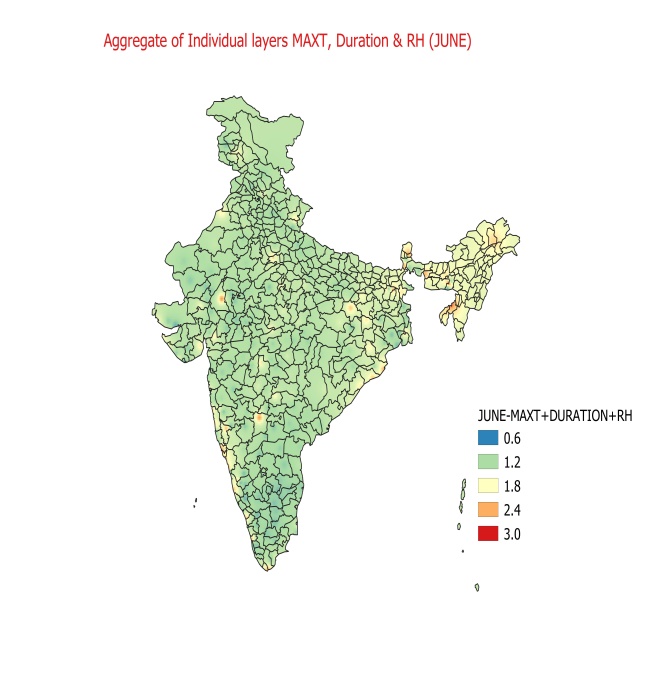 |
| 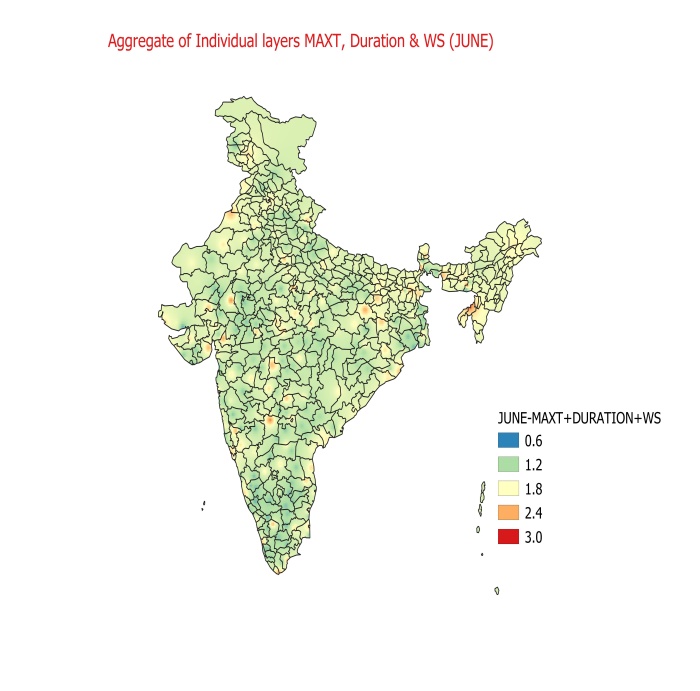 | 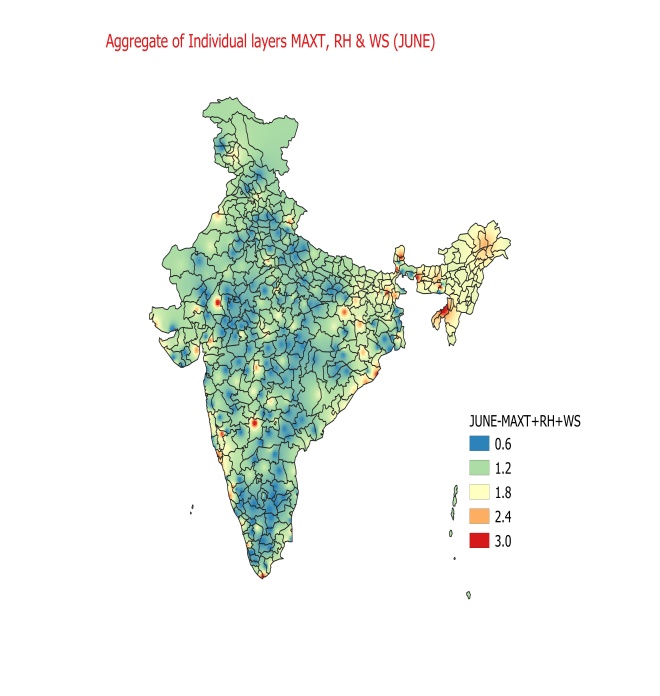 |
| 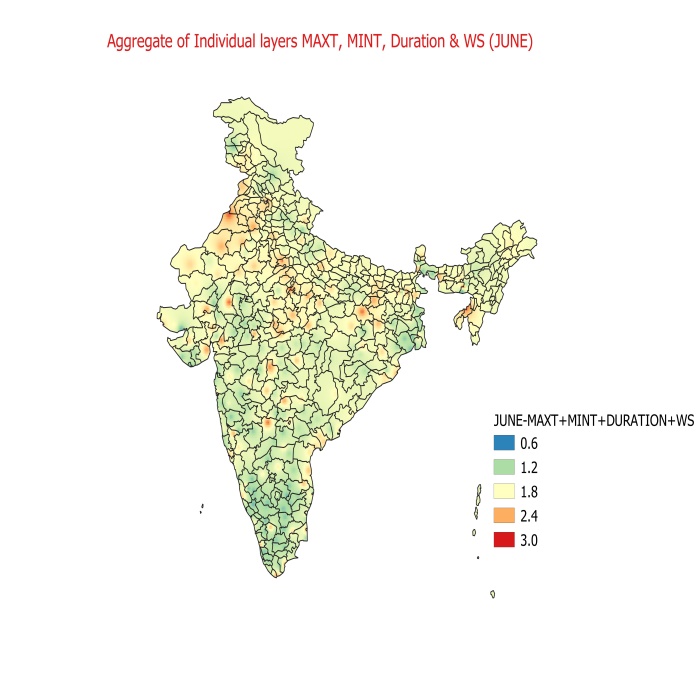 | 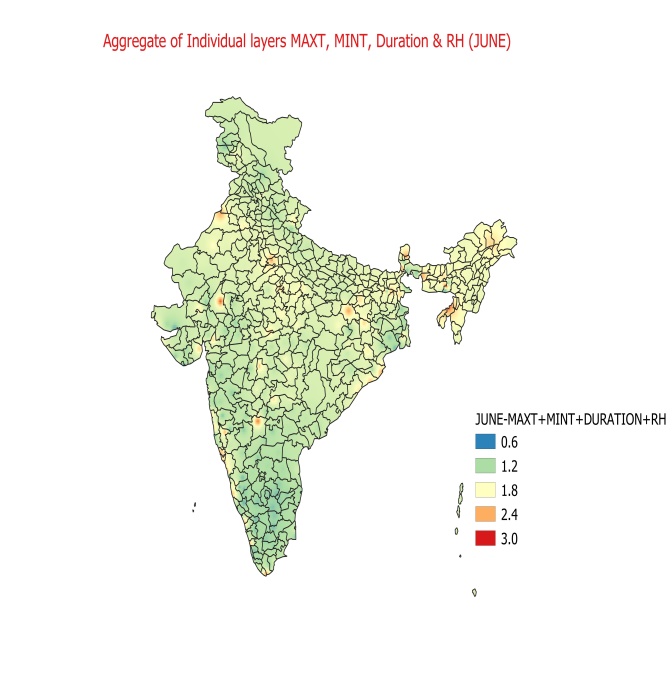 |
| Fig S4. June month mean daily weighted scores by aggregating different permutation and combination of meteorological parameters having influence on heat waves impact. | |

| 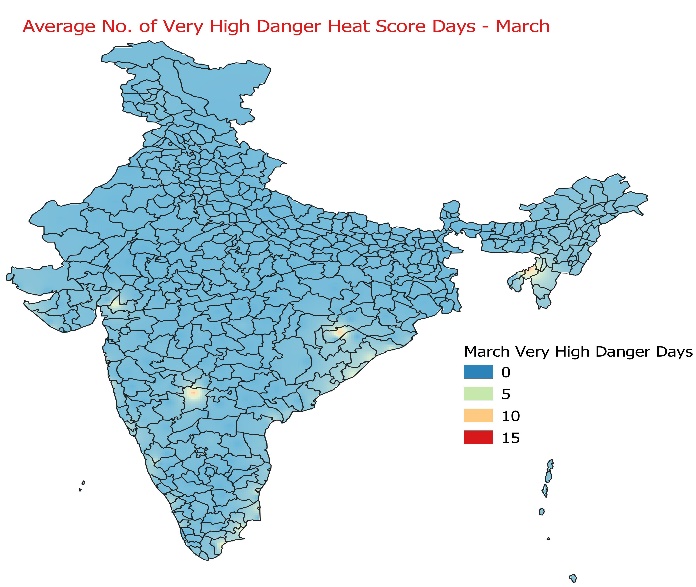 | 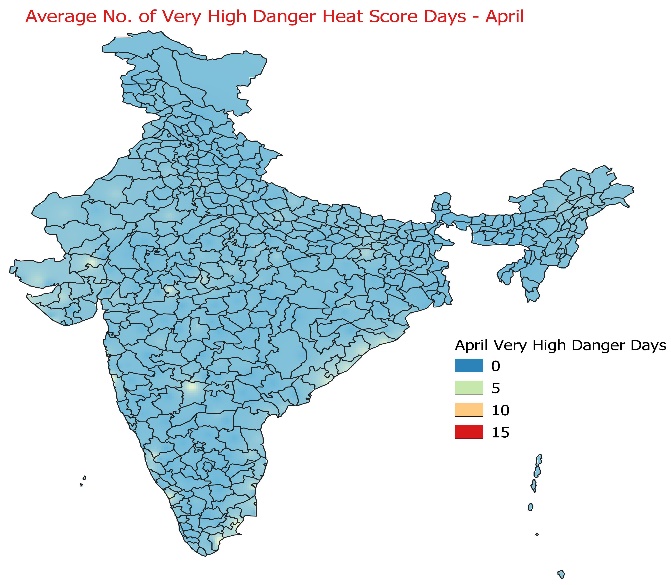 |
| --- | --- |
| 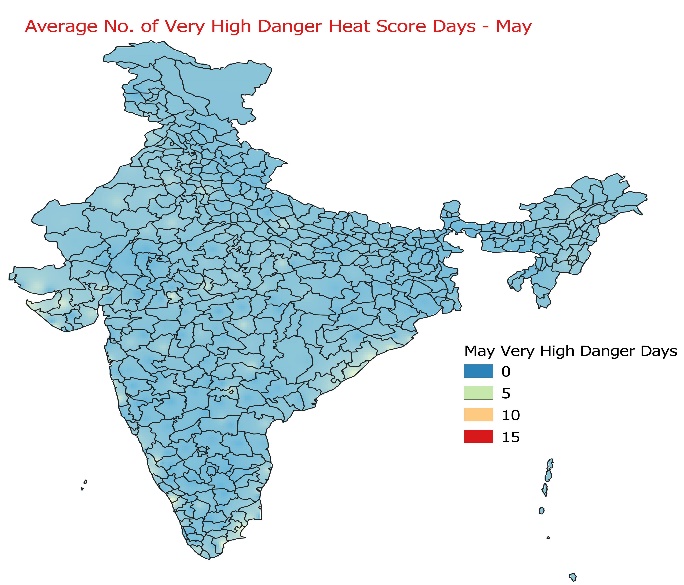 | 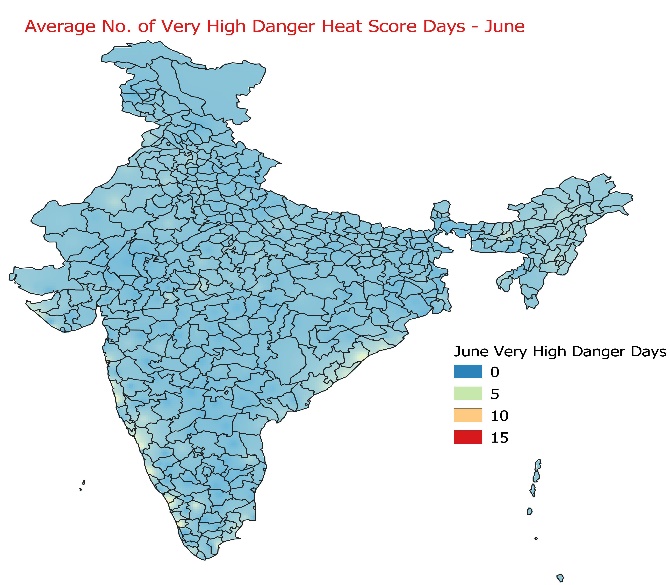 |
| Fig S5. Average number of days in the months of March to June under the category of Very High Danger level of Heat Hazard | |

| 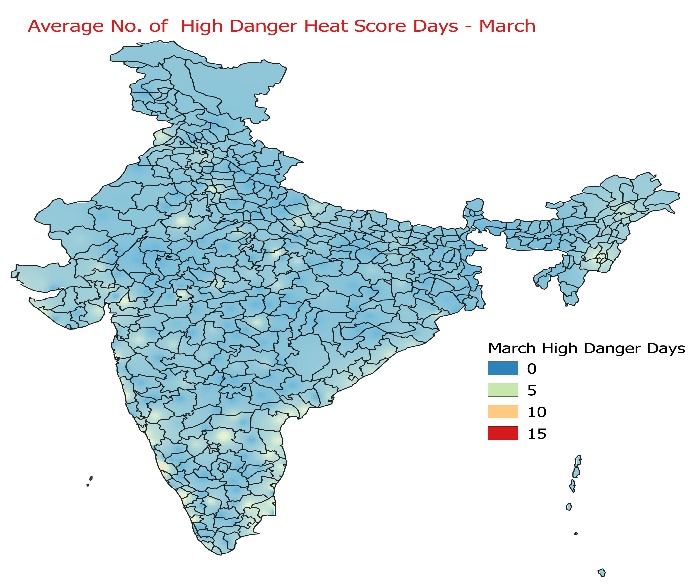 | 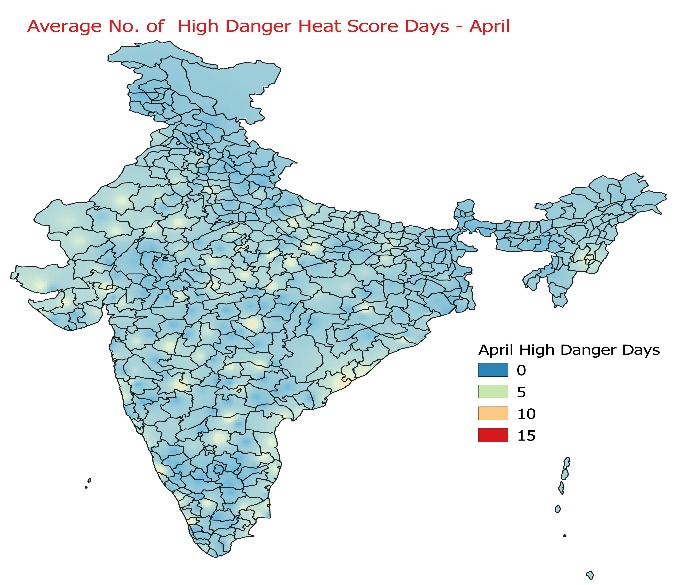 |
| --- | --- |
| 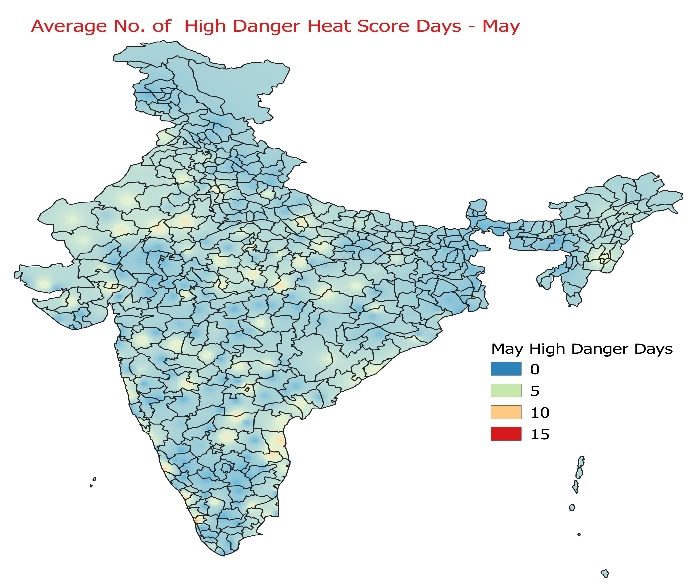 | 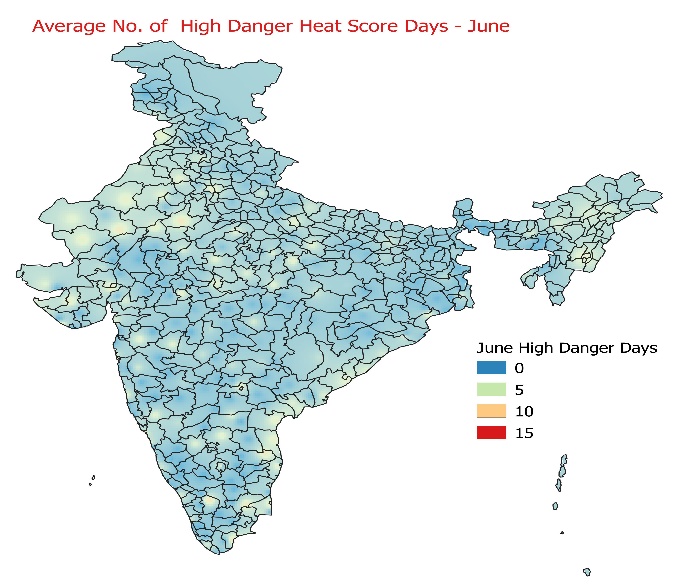 |
| Fig S6. Average number of days in the months of March to June under the category of High Danger level of Heat Hazard | |

| 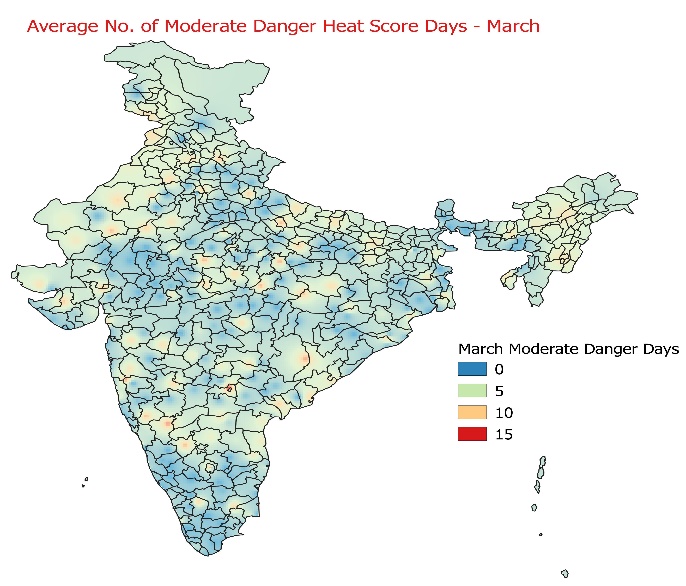 | 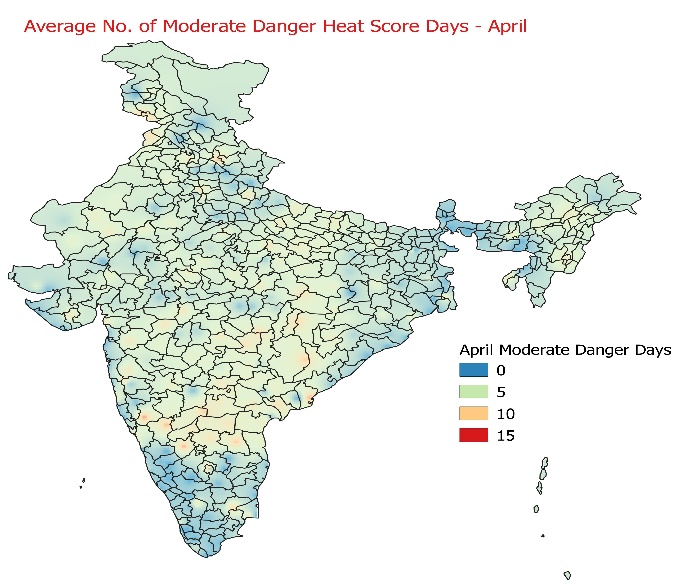 |
| --- | --- |
| 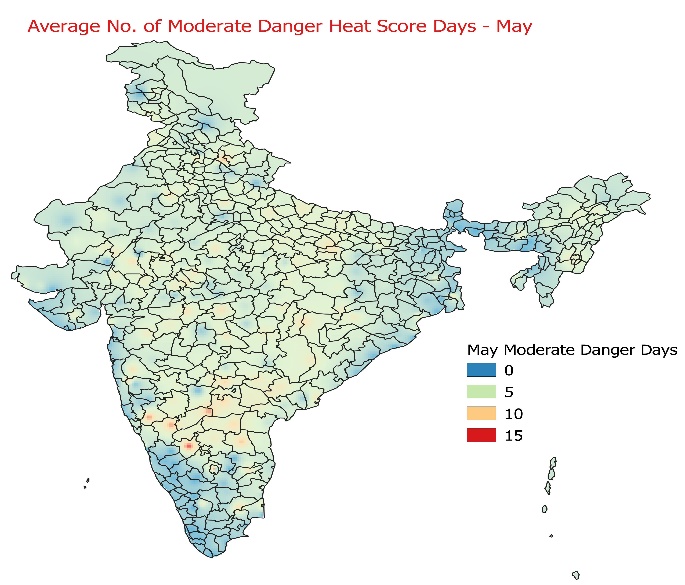 | 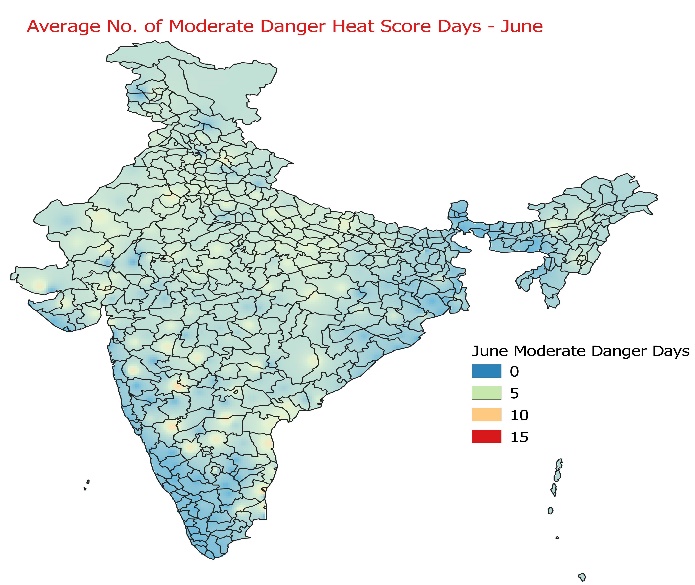 |
| Fig S7. Average number of days in the months of March to June under the category of Moderate Danger level of Heat Hazard | |
